# The human neuropsychiatric risk gene *Drd2* is necessary for social functioning across evolutionary distant species

**DOI:** 10.1038/s41380-023-02345-z

**Published:** 2023-12-19

**Authors:** Kevin G. O. Ike, Sanne J. C. Lamers, Soumya Kaim, Sietse F. de Boer, Bauke Buwalda, Jean-Christophe Billeter, Martien J. H. Kas

**Affiliations:** Groningen Institute for Evolutionary Life Sciences, University of Groningen, Groningen, The Netherlands

**Keywords:** Genetics, Neuroscience

## Abstract

The *Drd2* gene, encoding the dopamine D*2* receptor (D2R), was recently indicated as a potential target in the etiology of lowered sociability (i.e., social withdrawal), a symptom of several neuropsychiatric disorders such as Schizophrenia and Major Depression. Many animal species show social withdrawal in response to stimuli, including the vinegar fly *Drosophila melanogaster* and mice, which also share most human disease-related genes. Here we will test for causality between *Drd2* and sociability and for its evolutionary conserved function in these two distant species, as well as assess its mechanism as a potential therapeutic target. During behavioral observations in groups of freely interacting *D. melanogaster*, *Drd2* homologue mutant showed decreased social interactions and locomotor activity. After confirming *Drd2*’s social effects in flies, conditional transgenic mice lacking *Drd2* in dopaminergic cells (autoreceptor KO) or in serotonergic cells (heteroreceptor KO) were studied in semi-natural environments, where they could freely interact. Autoreceptor KOs showed increased sociability, but reduced activity, while no overall effect of *Drd2* deletion was observed in heteroreceptor KOs. To determine acute effects of D2R signaling on sociability, we also showed that a direct intervention with the D2R agonist Sumanirole decreased sociability in wild type mice, while the antagonist showed no effects. Using a computational ethological approach, this study demonstrates that *Drd2* regulates sociability across evolutionary distant species, and that activation of the mammalian D2R autoreceptor, in particular, is necessary for social functioning.

## Introduction

Most species form groups in which social interactions occur. This includes evolutionary distant species such as the vinegar fly, *Drosophila melanogaster* [1, 2], the mouse, *mus musculus* [3, 4], and humans [5]. Flies, mice and primates all show a preference for being in proximity to their conspecifics over being alone [6–8]. The proximity allows flies, mice and humans to interact and recognize individual group members. Indeed, mice sniff and flies touch with their legs individuals in their proximity, which respectively allows the nose of mice and the legs of flies -which harbor sensory neurons- to sense infochemicals carrying information about the identity and status of specific individual group members [9–12]. However, there is variation within species, where some individuals display a heightened or reduced tendency to join in group activity, a behavior termed sociability. Adjusting sociability level to environmental cues is a common adaptive behavior. For example, individuals withdraw from their group after exposure to (social) stress or sickness-inducing pathogens (see for examples [13, 14]). However, it becomes pathological when it is expressed inappropriately and/or excessively [15], standing in the way of normal social functioning.

Humans display exaggerated social withdrawal symptoms in a variety of psychiatric and neurological disorders, such as –Schizophrenia, Alzheimer’s disease and Major Depression, [16, 17]. This deprives patients from healthy social interactions and consequently impairs their quality of life [18]. A recent study showed that sociability scores are significantly lower in psychiatric patients compared to the general population [19]. In this same study, a human genome-wide association study (GWAS) found that sociability scores were correlated to variation in the gene encoding the dopamine D*2* receptor (*DRD2*) [19]. An independent GWAS that aimed at identifying genetic loci associated with Schizophrenia, a psychiatric disorder in which patients display social withdrawal [16], similarly identified *DRD2* [20]. Together this indicates that *DRD2* plays an important role in sociability.

The dopamine D2 receptor (D2R) is a G-protein coupled receptor expressed in both dopaminergic (i.e., autoreceptors) and non-dopaminergic neurons (i.e., heteroreceptors), exerting an inhibitory effect on neuronal excitability when activated by endogenous dopamine (DA) or synthetic agonists [21]. The D2R autoreceptor is responsible for the majority of autofeedback inhibition, decreasing both electrical activity of dopaminergic neurons and their DA synthesis and release (see for review [22]). In *D. melanogaster*, Dop2R is the homolog of D2-like receptors such as D2R (reviewed in [23]) and is expressed in both dopaminergic and non-dopaminergic neurons, as in vertebrates [24]. Dopamine has several important functions in the general control of behaviors. In mammals, the nigrostriatal dopaminergic system originating in the substantia nigra (SNR) has a central function in the control of motor output, and hence the proper execution of all behaviors. The mesolimbic dopaminergic system, originating in the ventral tegmental area (VTA) and projecting to the ventral striatum/nucleus accumbens (NAc), is key for the motivational and rewarding aspects of behavior. At the cellular and circuit level, motivational drive for virtually all behaviors is dependent on the concentration of extrasynaptic DA present in the striatum/NAc [25, 26]. Depletion or antagonism of DA in this area decreases the amount of work rodents are willing to spend to acquire a food reward [27].

Affiliative social interactions are rewarding to humans [28] and rodents [29]. Similar to their willingness to work for appetitive food rewards, rodents are also willing to work to obtain social rewards [30, 31]. Therefore, as DA increases willingness to work for a reward, higher DA concentration should also lead to an increase in sociability. Indeed, heightened dopamine levels in the cortex are correlated with heightened sociability between mouse strains [32]. Similarly, increased dopaminergic activity in the VTA relates to increased motivation to socially interact within the C57BL/6J strain [33]. Similar mechanisms are found in *Drosophila*, where Dop2R regulates locomotor behavior [34] and plays a role in associative learning [35]. Dopamine is also linked with appetitive memory formation and social interactions in *Drosophila* [36–38].

Not only is dopaminergic functioning correlated with social personality traits, social stressors can also impact the dopaminergic system, both acutely [39, 40] and long-term [41, 42]. In rodents, long term social stress due to social isolation, leads to a decrease in *Drd2* expression in the medial prefrontal cortex (mPFC) and the NAc shell, which coincides with impaired social discrimination and increased aggression [43]. Pharmacological studies in rats making use of Quinpirole and Sulpiride, respectively an agonist and antagonist of D2R, have shown that manipulation of D2R leads to changes in social behavior; where the agonistic action of Quinpirole decreases social behavior, antagonism by Sulpiride increases it [44]. This suggests that *Drd2* can be linked to sociability and that a decrease in expression might induce a hyper-sociable phenotype, at least in rodents.

This study aims to causally link *Drd2* to sociability, assess its evolutionary conserved function and assess its mechanism as a potential therapeutic target. To causally link *Drd2* to sociability a classical whole-body genetic knockout will be performed in *D. melanogaster* as this approach allows for efficient screening for any social effect related to *Drd2*. Since altered DA levels in the brain have shown to affect social space, we expect to find altered social behavior in these flies.

If *Drd2* influences sociability in *D. melanogaster*, it justifies studying this gene in mice, a species that is an established mammalian model for translation to humans [45]. However, in mice, whole body and whole brain deletions of *Drd2* are associated with notable decreases in body weight and other physiological characteristics [46–48] that will be detrimental to the employed methods of measuring sociability in a semi-natural environment. Thus, the general *Drd2* knockout will not be used in mice and only a cell-type specific (conditional KO) approach will be employed to elucidate the D2R-expressing cell types that plays a role in sociability. The cre/lox system will be utilized to create a conditional knockout model in mice, allowing for deletion of *Drd2* in specific cell types. Here, *Drd2* is deleted in dopaminergic or serotonergic cells, making use of the *DAT*^*IRES*^*-cre* [49, 50] and *ePet-cre* [51] lines respectively. Dopamine D2 receptor expression in dopaminergic cells (i.e., the D2R autoreceptor) is central to the efficacy of most drugs effective against SZ symptoms [52, 53], which includes social withdrawal [54]. Deleting the autoreceptor removes the negative feedback of DA on dopaminergic neurons, thereby increasing DA release upon activation of these neurons [50]. These autoreceptor KO mice display increased locomotor activity and increased motivation for food reward [50]. As increased dopaminergic activity correlates with an increase in sociability, the autoreceptor KO mice are predicted to display a socially enhanced phenotype. The serotonergic D2R heteroreceptor was chosen, as deletion of D2R on serotonergic cells has been implicated in inter-male aggression and locomotor activity in mice, while silencing the D2R-expressing serotonergic cells increases these behaviors [55]. In general, modulation of serotonergic activity has clear social consequences (see for review [56]), making the serotonergic heteroreceptor a clear target for social behavior. As dopamine would normally inhibit D2R expressing serotonergic neurons [55] and silencing (i.e., an extreme form of inhibition) these neurons cause the phenotype mentioned above, the heteroreceptor KOs are hypothesized to show the opposite phenotype: decreasing social behaviors and locomotor activity.

The multiple model species approach will allow the assessment of the evolutionary conserved function of *Drd2*. Altered social behavior, after manipulating *Drd2* in both flies and mice, would indicate functional evolutionary conservation of *Drd2*. Previous research points at the functional conservation of disease-related genes. Approximately 99% of murine genetics can be related to humans, with ~80% being direct orthologs [57]. When comparing human genes linked to diseases to corresponding genes in mice with a similar phenotype, there is an overlap of ~70% [58]. In addition, about 77% of human disease-associated genes can be found in *D. melanogaster*, from which about 10% are associated with neurological pathologies, including psychiatric disorders [59]. This suggests that the genetic risk factors related to social impairments, *as Drd2*, might also be similar in evolutionary distant species.

After confirming social effects of germline *Drd2* manipulation in mice, short-term selective pharmaceutical manipulation of D2R is performed to study the acute effects of DR2 signaling on sociability and to highlight the therapeutic potential for this receptor to target pathological social withdrawal. The behavioral effects of both a pharmacological agonist (Sumanirole) and an antagonist for D2R (L-741,626) are assessed, which have previously been shown to transiently induce behavioral changes [60, 61].

Together, the results of this study will provide insight into an evolutionary conserved molecular mechanism that regulates sociability and further aims to confirm *Drd2* as a potential therapeutic target for social withdrawal.

## Methods

### Animal rearing and strains

#### Drosophila melanogaster

strains were raised in food medium containing: agar (10 g/L), glucose (167 mM), sucrose (44 mM), yeast (35 g/L), cornmeal (15 g/L), wheat germ (10 g/L), soya (10 g/L), molasses (30 g/L), propionic acid (5 ml of 1 M) and Tegosept (2 g in 10 ml ethanol) in bottles (6 oz square bottom, polypropylene) or vials (2.3 cm × 9.3 cm, polystyrene) at ±25°C under a 12:12 light/dark cycle.

The following fly strains were used: *Dop2R*^*Δ2*^*;+;+* (stock#: 78796) and *Oregon-R* (*OR*) stocks were obtained from the Bloomington stock center. *Dop2R*^*Δ2*^*;+;+* was placed in an *OR* background by outcrossing 5 times to *w*^*1118*^*,OR;OR;OR* (*w*^*1118*^ previously outcrossed 10 times to *OR*). The resulting offspring were crossed to *OR* stock. *w*^*1118*^ was selected against and PCR used to track the *Dop2R*^*Δ2*^ mutant allele and generate *Dop2R*^*Δ2*^*;OR;OR* mutants. To assess the effect of a whole-body knockout of *Drd2* the *Dop2R*^*Δ2*^*;OR;OR* mutant was compared to OR wildtype flies.

#### Mice

were maintained under a 12:12 light/dark cycle, controlled temperature (21 ± 1 °C) and humidity (55 ± 5 %) and with ad libitum access to food and water in Makrolon type 3 cages on Aspen chip bedding (Lignocel BK8/15). All mice were weaned at 21-28 days of age and kept under these standard housing conditions in groups of 4 males up until the start of the experiment, between 11-18 weeks of age.

Transgenic mouse lines were obtained from The Jackson Laboratory (Bar Harbor, Maine, USA) via Charles River Europe (Den Bosch, The Netherlands), Drd2-loxp (B6.129S4(FVB)-Drd2tm1.1Mrub/J; JAX stock #020631; [50]), ePet-cre (B6.Cg-Tg(Fev-cre)1Esd/J; JAX stock #012712; [51]) and DAT^IRES^-cre (B6.SJL-Slc6a3tm1.1(cre)Bkmn/J; JAX stock #006660; [49]). Homozygous Drd2-loxp mice were crossed with both hemizygous ePet-cre and hemizygous DAT^IRES^-cre animals. Subsequent offspring was further crossed to acquire the experimental lines in which *Drd2* is deleted specifically in either dopaminergic (Drd2-loxp x DAT^IRES^-cre, autoreceptor KO; after Bello et al. (2011) [50] or serotonergic (Drd2-loxp x ePet-cre, heteroreceptor KO) cells. Wildtype (WT) C57BL/6j animals originated from our laboratory colony, but were originally obtained from Charles River Laboratories (Germany, stock #027). All animal care was executed according to the local rules set by the ethical authorities.

### D. melanogaster social assay

**Fig. 1:**
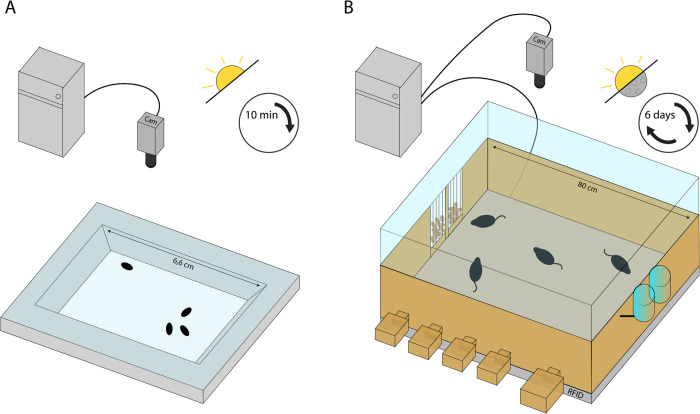
Schematic representation of experimental setups. Both flies and mice were recorded using a digital camera above a rectangular arena in which 4 animals could freely interact. Flies interacted in a plexiglass arena with sloped walls and a low glass ceiling to ascertain an upright position and visibility during the 10 min recordings in the light phase (**A**). Mice interacted in a plastic arena, with multiple nests attached to it and ad-libitum access to food and water (**B**). The ceiling consisted of transparent plexiglass to ensure visibility. Data from both the RFID-receiver, below the arena, and the digital camera were collected, stored and combined in the attached computer. Recordings of mice behavior were conducted around the clock for 6 days, in both the light and dark phase.

Virgin flies were collected using CO_2_ anesthesia on the day of eclosion and were kept in same-sex groups of 15-25 flies per vial for 5-7 days. Groups of 4 flies were introduced in a 66 x 40 x 3 mm plexiglass arena where they could freely move and interact for 10 minutes. The arena is milled out of plexiglass with slopes as walls to ensure optimized video tracking of the flies (modeled after the fly bowl; Simon and Dickinson (2010) [62] and topped with a removable glass plate (69 × 69 mm) (Fig.1A). Arenas and glass were cleaned with 70% ethanol between experiments. Ten-minute-long videos were captured with a Raspberry Pi + High-Quality camera module with 16 mm tele lens. TGrabs and Trex software [63] was used to analyze the videos. First, TGrabs was used for preliminary identification of the flies in each video, whereafter TRex was used for tracking fly identity and partly analyzed the data. A custom-written python script (https://github.com/SanneLamers/FlySocial) used the data generated by TRex to identify social interactions and locomotor activity. Social interactions between two flies were quantified as in Schneider et al., (2012) [64]: (1) the angle between the long axis of fly 1’s body length and the center of fly 2 should be less than 90^o^, (2) the distance between the center of the two flies was less than or equal to two body lengths, and (3) these conditions are maintained for at least 1.5 seconds. Thus, what we define as a social interaction in this manuscript is the behavioral analogue of mouse sniffing.

### Mouse *Drd2* genetic studies

To study gene function conservation of *Drd2* in mice, specific *Drd2* gene deletions were generated for the dopaminergic autoreceptor and serotonergic heteroreceptor (see above), and subsequently tested in a semi-natural observational arena. Prior to the experiment, male mice were housed in groups of 4 in which the genotype composition was akin to what they would be exposed to during the experiment. One week before the onset of the experiment, C57Bl/6J animals were injected with a Radio-frequency identification (RFID) chip (8 mm × 2 mm). RFID-chip was injected subcutaneously in the dorsal/caudal part of the animal under anesthesia (i.e., Isoflurane ~2%). On experimental day one, 4 mice, taken from 4 different home-cages, were placed in an observational arena (Fig.1B) and left undisturbed for 6 days (see [65]), after which animals were sacrificed. Each “treatment” colony consisted of four animals, two *cre*-expressing animals, one of those homozygous for *Drd2-loxp* (KO) and one heterozygous for the gene (HET), and two animals not expressing cre (WT/x). The “control” colonies also consisted of two *cre*-expressing animals, but now one wildtype for *Drd2-loxp* (WT) and one heterozygous for the gene (HET), similarly including two animals not expressing cre (WT/x) (Table 1). In these colonies the KO and WT animals served as experimental animals, while all others provided social stimulus for these two groups. Controls (WT) for KO’s were always wildtype animals expressing the same cre-gene (i.e., *DAT*^*IRES*^*-cre* or *ePet-cre*). This design allows for direct comparison of KO and WT animals across colonies, as experimental animals from both groups are exposed to a similar social environment. Behavior was automatically annotated using RFID-assisted SocialScan (CleverSys Inc., Reston, USA; see supplementary table S1).

**Table 1:** Organization of colonies for Drd2 genetic studies in mice.

Treatment colony (*n* = 7)			Control colony (*n* = 7)		
Name	***Cre***	***Drd2-loxp***	**Name**	***Cre***	***Drd2-loxp***
Receptor knock-out (KO)	+/-	+/+	Receptor wildtype (WT)	+/-	-/-
Receptor heterozygote(HET)	+/-	+/-	Receptor heterozygote (HET)	+/-	+/-
*Cre* wildtype (WT/x)	-/-	+/+ or +/- or -/-	*Cre* wildtype (WT/x)	-/-	+/+ or +/- or -/-
*Cre* wildtype (WT/x)	-/-	+/+ or +/- or -/-	*Cre* wildtype (WT/x)	-/-	+/+ or +/- or -/-

Each treatment colony consists of one animal that is hemizygous for the Cre-gene and homozygous for Drd2-loxp (receptor knock-out; KO), one animal that is hemizygous for the Cre-gene and heterozygous for Drd2-loxp (receptor heterozygote; HET) and two animals that do not possess the Cre-gene and are either homozygous, heterozygous or wildtype for Drd2-loxp (Cre wildtype; WT/x). Control colonies consist of one animal that is hemizygous for the Cre-gene and wildtype for Drd2-loxp (receptor wildtype; WT), one animal that is hemizygous for the Cre-gene and heterozygous for Drd2-loxp (receptor heterozygote; HET) and two animals that do not possess the Cre-gene and are either homozygous, heterozygous or wildtype for Drd2-loxp (Cre wildtype; WT/x). Genotypes are shown as symbols where +/+, +/- and -/- represent homozygous, heterozygous/hemizygous or wildtype expression of the gene, respectively.

### Mouse D2R pharmacological studies

To evaluate the effects of acute manipulation of D2R, the behavior of groups of wildtype mice was measured longitudinally in semi-natural observational arenas after injection of either a selective D2R agonist Sumanirole (3 mg/kg, dissolved in saline; Sumanirole-Maleate; Sigma-Aldrich) or a selective D2R antagonist L-741,626 (1 mg/kg, dissolved in a 5% Ethanol/saline solution; L-741,626; Sigma-Aldrich). Solutions were prepared daily. One week before the onset of the experiment, C57Bl/6J males were injected with a RFID-chip. The RFID-chip was injected subcutaneously in the dorsal/caudal flank of the animal under anesthesia (i.e., Isoflurane ~2%). On experimental day one, 4 unacquainted C57BL/6J mice were placed in a semi-natural observational arena (see [65]) and left undisturbed for two days. On the third day, one of the animals, from each arena, was randomly selected, weighed, injected with either the drug of choice or its vehicle and placed back in the arena just before the beginning of the dark phase. The three other animals of each arena were also weighed and placed back. Every third day after a day of injection these steps were repeated until all four animals were injected with either the drug or its vehicle. Each arena had a “twin” arena in which the treatment was counterbalanced, meaning that when an animal in one arena was injected with a drug the mouse in another adjacent arena (~75 cm away) was injected with its vehicle, accounting for possible order effects of the drug treatment. On day 15 the animals were sacrificed, ending the experiment. In total 6 arenas were tested for each drug (*n* = 12). Behavior was automatically annotated using RFID-assisted SocialScan (CleverSys Inc., Reston, USA).

### Statistical analysis

Data regarding the manipulation of *Dop2R*^*Δ2*^ in *D. melanogaster* were summed over the 10-minute recording per arena containing a group of 4 animals and subsequently analyzed per sex in RStudio (version 1.4.1717), making use of the packages *readxl*, *car*, *ggplot2* and *cowplot* [66–69] and graphed using Graphpad Prism (Version 9.3.1 (350)). Activity, measured as average speed, was assessed by means of a linear model. Number of interactions and the average durations per interactions violated the criteria for normality based on the Shapiro-Wilk test (respectively, W = 0.69081, *p*-value = 6.902e-08; W = 0.6638, p-value = 2.604e-08) and were tested using generalized linear models, Number of interactions was based on a Poisson distribution and average duration per interactions on a gamma distribution.

Analysis of the data regarding genetic interventions in mice and pharmacological interventions was performed by means of generalized additive models (GAM) in RStudio (version 1.4.1717), making use of the packages *readxl*, *plyr*, *nlme*, *mgcv*, *itsadug*, *ggplot2*, *extrafont* and *cowplot* [67–78]. Data of social behaviors (i.e., time spent approaching/sniffing conspecifics) was collected as cumulative duration of time spent on the behavior towards any of the conspecifics in their respective arena in bins of 1 hour. Subsequently, data was log-transformed and then modeled based on a gaussian distribution, where the statistical significance of the effect of genetic manipulation was assessed over time using a GAM for repeated measures. Time while in one of the nests was used as a covariate to correct for the inability to observe social behavior while in the nest. This was followed by inspection of the difference plot for comparison of KO and WT at specific timepoints. Activity, in the form of total distance moved, was modeled based on a quasipoisson distribution. Data regarding pharmacological manipulation in mice was analyzed by subtracting the cumulative duration of time spent on the behavior on the 24 hours before (i.e., baseline) from the time spent on the behavior on the 24 hours directly after treatment for each corresponding hour. The same was done for total distance moved. For these increases from baseline the statistical significance of the effect of pharmacological manipulation was modeled over time using a GAM for repeated measures based on a gaussian distribution, followed by inspection of the difference plot for comparison of treated and control animals at specific timepoints. The predicted data based on these models is presented in figures 3–6.

## Results

### Dop2R mutation reduces social behavior and locomotor activity in flies

Drosophila social behavior includes leg touching that has a well-documented function in recognition [9, 11, 12]. Legs harbor chemosensory neurons that sense pheromones on the body of the flies they are interacting with. We used an automated tracking system set to detect when flies are in a position to touch another fly, and defined this as one social interaction [64, 79].

**Fig. 2:**
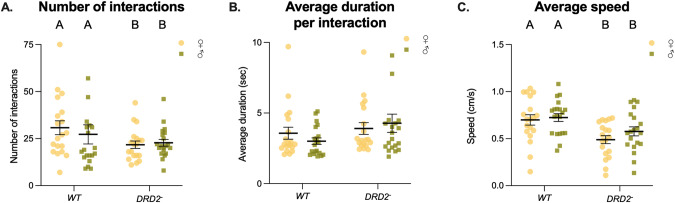
Dop2RΔ2 KO in male Drosophila melanogaster: Social behavior and locomotor activity. Data is presented as means with SEM showing the total number of interactions (**A**), the average duration per interaction (B) and total distance moved in cm (**C**) on the Y-axis based on 10-minute observations, with the genotype on the X-axis. Groups of females are shown in yellow dots (*n* = 19-20) and male in green squares (*n* = 19–20). Each dot or square represents an individual group of 4 flies, the black line represents the mean with SEM. Significant differences between groups are indicated with different letters.

Social behavior and locomotor activity were assessed in flies with a null mutation of *Dop2R*^*Δ2*^. Mutant flies showed significantly fewer social interactions (*χ²* = 35.34, *df* = 2, sex: *p* = 0.28, genotype: *p* = 5.85e-09; Fig. 2A). The average duration of social interactions did not significantly differ from wildtypes (*χ²* = 35.34, *df* = 2, sex: *p* = 0.828, genotype: *p* = 0.0719; Fig. 2B). *Dop2R*^*Δ2*^ deficient mutants also displayed a significantly lower average speed over the 10-minute observation compared to wildtype flies (*F*_2,75_ = 7.906, sex: *p* = 0.233, genotype: *p* = 0.0003; Fig. 2C).

### Deletion of *Drd2* in dopaminergic cells increased social behavior, but reduced activity

**Fig. 3:**
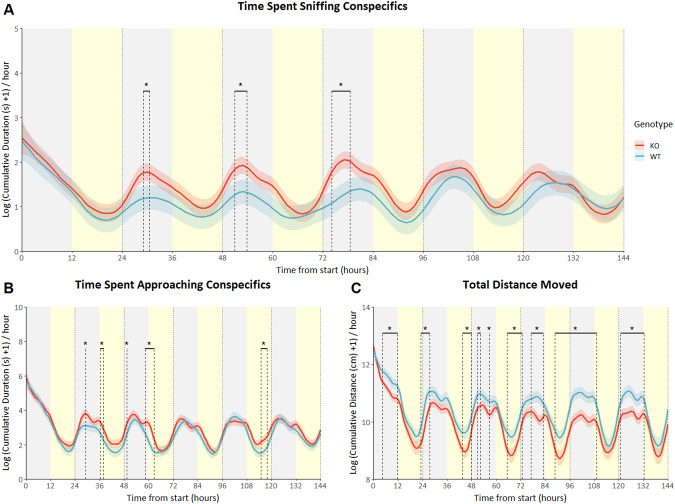
Drd2 autoreceptor KO mice: predicted social behavior and locomotor activity based on modeled data. Data is presented as the logarithm of cumulative time spent sniffing conspecifics in seconds (**A**), the logarithm of cumulative time spent approach conspecifics in seconds (B) and the logarithm of total distance moved in cm (**C**) on the Y-axis (mean ± SEM) based on 1-hour bins, with time from the start of the experiment on the X-axis in hours. Autoreceptor knockout animals (*n* = 7) are shown as the red lines, wildtype animals (*n* = 7) are shown as blue lines. Yellow shading behind the graph indicates the light phase, where gray shading indicates the dark phase. *n* = 7; * *p* < 0,05 based on difference plots after modeling the data with a GAM. (see figures S1-S5 for individual data points and means of observed values).

Mice with specific deletion of either the D2R autoreceptor or serotonergic heteroreceptor were generated to assess the consequences of long-term specific *Drd2* deficiency. Animals in which the D2R autoreceptor was selectively knocked out showed a significant increase in social sniffing behavior when compared to wildtype animals in a semi-natural observational arena in which they could freely interact with their respective conspecifics during certain times of the day (genotype*time interaction F_(23.875_, _1897.726)_ = 4.47; *p* < 0.001), there was no overall effect (p = 0.122). Specifically, in the middle of the dark phase of days 2–4 (Fig. 3A), KOs spent more time sniffing their conspecifics than WT animals. Autoreceptor KOs spent significantly more time approaching conspecifics in general (*p* = 0.038). During days 2 and 3, increases are found during both the dark phase and the beginning of the light phase. On day 5 KOs showed more approach behavior during the light phase only (genotype*time interaction F_29.101_, _1898.159)_ = 6.25; *p* < 0.001; Fig. 3B). Activity, measured as total distance moved, was shown to be significantly decreased in KOs during most of the time the animals spent in the observational arena (*p* < 0.001). This effect was seen primarily in the dark phase, the phase in which mice in general display most of their activity. The relative decrease in activity started after the first few hours after entering the arena, lasting for the latter two thirds of the dark phase at day 1 and beginning again just before the start of the dark phase of the second day, lasting 4 hours. At days 3–6, this increase in activity continued. Here the decrease in distance moved shifted from the beginning of the dark phase at day 3, to the end of the dark phase at day 4 and lasted the full dark phase of days 5 and 6. Considering the light phase, at days 2–4 the KOs showed a decrease in activity at the end of each light phase (genotype*time interaction F_(32.396_, _1907.132)_ = 5.72; *p* < 0.001; Fig. 3C).

**Fig. 4:**
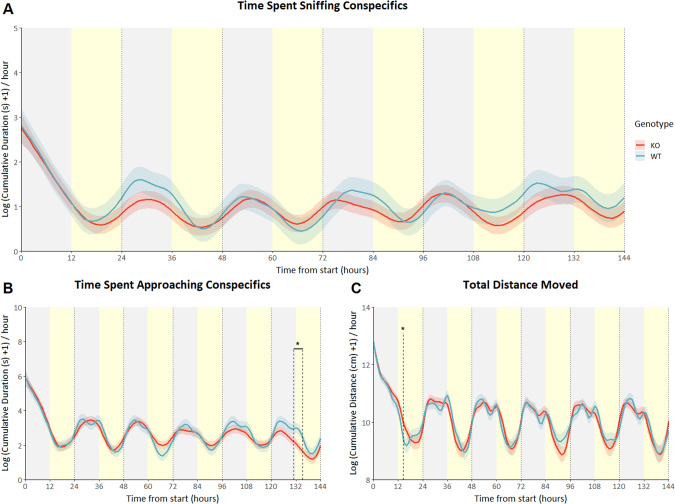
Serotonergic Drd2 heteroreceptor KO mice: predicted social behavior and locomotor activity based on modeled data. Data is presented as the logarithm of cumulative time spent sniffing conspecifics in seconds (**A**), the logarithm of cumulative time spent approach conspecifics in seconds (**B**) and the logarithm of total distance moved in cm (**C**) on the Y-axis (mean ± SEM) based on 1-hour bins, with time from start of the experiment on the X-axis in hours. Heteroreceptor knockout animals (*n* = 6) are shown as the red lines, wildtype animals (*n* = 8) are shown as blue lines. Yellow shading behind the graph indicates the light phase, whereas gray shading indicates the dark phase. *p < 0,05 based on difference plots after modeling the data with a GAM. (see figures S6–S10 for individual data points and means of observed values).

No overall effects on any of the behaviors were found when knocking out *Drd2* selectively in serotonergic neurons (sniffing: *p* = 0.385, approaching: *p* = 0.53, total distance moved: *p* = 0.972). However during a very limited number of time points a decrease in social behaviors was observed. Regarding sniffing behavior, heteroreceptor KOs showed a significant decrease over time when compared to wildtype animals (genotype*time interaction F_(15.706_, _1821.834)_ = 2.13; *p* < 0.01; Fig. 4A). However, this effect could not be pinpointed to specific times. Heteroreceptor KOs also showed a slight decrease in approach behavior, apparent at the end of the dark phase at day 6 and continuing into the beginning of the light phase (genotype*time interaction F_(21.039_, _1846.899)_ = 4.60; *p* < 0.001; Fig. 4B). At the beginning of the light phase at day 1 KOs showed a short increase in total distance moved, a measurement of activity (genotype*time interaction F_(33.291_, _1838.534)_ = 5.89; *p* < 0.001; Fig. 4C).

### D2R agonist Sumanirole decreases social behavior directly after injection

**Fig. 5:**
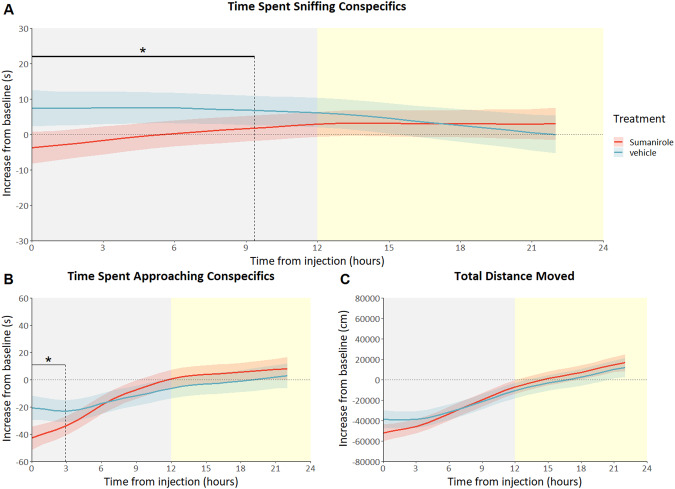
Mice injected with the D2R agonist Sumanirole: predicted social behavior and locomotor activity based on modeled data. Data is presented as the increase in time spent approaching conspecifics compared to baseline (**A**), the increase in time spent sniffing conspecifics compared to baseline (**B**) and the increase in total distance moved compared to baseline (**C**) on the Y-axis (mean ± SEM) based on 1-hour bins, with time from injection on the X-axis in hours. Sumanirole-treated animals (*n* = 9) are shown as the red lines, vehicle-treated animals (*n* = 9 s) are shown as blue lines. Yellow shading behind the graph indicates the light phase, whereas gray shading indicates the dark phase. *p < 0,05 based on difference plots after modeling the data with a GAM. (see figures S11-S13 for individual data points and means of observed values).

Wildtype C57Bl/6j were injected with either a D2R agonist or antagonist, Sumanirole and L-741,626 respectively, to demonstrate the effects of short-term D2R manipulation in wildtype mice. Wildtype mice injected with Sumanirole (3 mg/kg), a selective D2R agonist, showed a clear decrease in social behavior relative to the day before the injection, when compared with the vehicle-treated controls. Sumanirole treated mice spent significantly less time approaching their conspecifics during the first 3 hours following treatment (treatment *time interaction F_(2.620_, _363.912)_ = 3.79; *p* < .01; Fig. 5A) and showed a significant decrease in sniffing behavior during the first 9 hours after injection, when compared with vehicle-treated controls (treatment *time interaction F_(1.002_, _369.557)_ = 4.86; *p* = 0.028; Fig. 5B). Overall, total distance moved appeared not to be affected by the treatment (treatment *time interaction F_(2.049_, _362.716)_ = 2.00; *p* = 0.172; Fig. 5C).

**Fig. 6:**
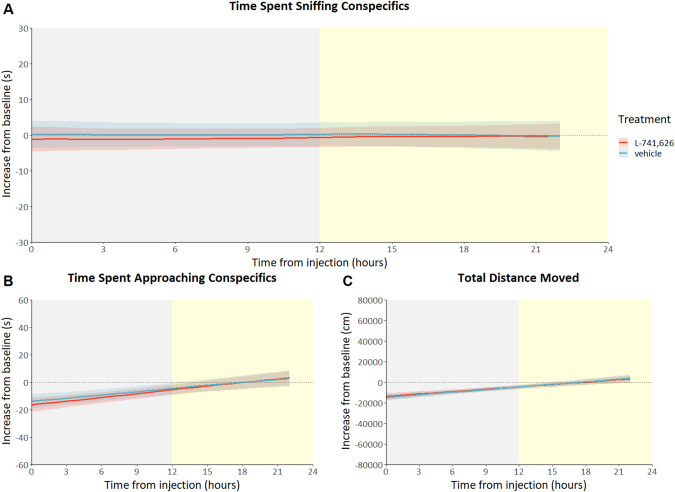
Mice injected with the D2R antagonist L-741,626: predicted social behavior and locomotor activity based on modeled data. Data is presented as the increase in time spent approaching conspecifics compared to baseline (**A**), the increase in time spent sniffing conspecifics compared to baseline (**B**) and the increase in total distance moved compared to baseline (**C**) on the Y-axis (mean ± SEM) based on 1-hour bins, with time from injection on the X-axis in hours. L-741,626 treated animals (*n* = 8) are shown as the red lines, vehicle-treated animals (*n* = 9) are shown as blue lines. Yellow shading behind the graph indicates the light phase, where gray shading indicates the dark phase. (see figures S14–S16 for individual data points and means of observed values).

Treatment with L-741,626 (1 mg/kg), a selective D2R antagonist, neither increased or decreased social behavior or activity, when comparing L-741,626 treated with vehicle-treated animals. No effect was found on approach behavior (treatment *time interaction F_(1.000_, _297.287)_ = 0.07; p = 0.796; Fig. 6A) or time spent sniffing (treatment *time interaction F_(1.000_, _293.220)_ = 0.04; p = 0.837; Fig. 6B). While time did significantly affect total distance moved (F_(1.003_, _302.321)_ = 10.86; *p* < .01; Fig. 6C), there was no effect of treatment (treatment*time interaction F_(1.000_, _302.321)_ = 0.04; *p* = 0.843; Fig. 6C).

## Discussion

This study shows that the human neuropsychiatric risk gene *DRD2* is necessary for social functioning across evolutionary distant species. We found that both insects (i.e., *Drosophila melanogaster*) and mammals (i.e., *mus musculus*) express altered sociability following genetic deletion of their *DRD2* gene homologues. Through genetic and pharmacological interventions in conditional transgenic and wildtype mice respectively, our study shows that activation of the mammalian autoreceptor is necessary for social functioning. These findings reveal causality for *DRD2’s* conserved gene function in sociability and provide a potential mechanism of action for targeting pathological social withdrawal in neuropsychiatric disorders.

From the data generated in *D. melanogaster*, it is clear that long-term whole-body *Dop2R*^*Δ2*^ deletion impacts sociability, as *Dop2R*^*Δ2*^ mutant flies display a decrease in a number of social interactions. However, general locomotor activity shows a similar decrease, as shown before by Draper et al. (2007) [34]. This indicates that the effects of *Dop2R*^*Δ2*^ deletion in flies is not confined to social behavior. It is therefore not clear whether the decrease in sociability is a direct effect of whole body *Dop2R*^*Δ2*^ deletion or if the decrease in locomotor activity led to a decrease in the number of interactions. However, if the decrease in number of interactions would be a direct effect of the decrease in locomotion, an increase in the average duration of the interaction in *Dop2R*^*Δ2*^ deficient flies could be expected. This would indicate that the flies would move less while interacting, leading to the observed effects on social behavior. However, the lack of an effect on the average duration of social interactions suggests a direct effect of whole-body *Dop2R*^*Δ2*^ deletion in flies on sociability.

In contrast to the decreased social behavioral effects of general *Dop2R*^*Δ2*^ deletion in *D. melanogaster*, an increase in sociability was observed after specifically deleting the D2R autoreceptor in mice. Similar to flies, however, mice displayed a decrease in locomotor activity. While *the deletion of DRD2 homologues* has species-specific effects in the relationship and direction of sociability and motor activity levels in flies and mice, our findings do show that deletion consistently influences sociability in both species.

In mice, deleting the D2R autoreceptor impairs negative feedback from locally released dopamine on dopamine neurons and hence leads to increased dopamine neuron excitability and augmented DA release [50]. Behaviorally, D2R autoreceptor KO mice were previously shown to exhibit enhanced motivation for rewarding properties of food and drugs of abuse [50]. Given that DA participates in reward-guided behavior [25, 26], engaging in social interactions has rewarding properties [29] and increases in dopamine correlate with increases in sociability [32], an increase in sociability is similarly expected here. Indeed, this is what is observed in the autoreceptor KO animals. During the first days, when social structures are formed, there is a distinct increase in sociability, measured as time spent sniffing and approaching conspecifics, during the dark/night phase (Fig. 3A-B). During this phase mice are generally known to be most active, which is demonstrated by their clear circadian rhythmicity in locomotor activity (Fig. 3C). Activity is also visibly affected by autoreceptor deletion; however, the direction of the effect contrasts that on sociability and remains after the effects on sociability wane. Previous research has shown an increase in locomotor activity when employing the same D2R autoreceptor KO model, though this increase was found in a conventional test where animals are transferred to the test environment [50]. During the first hours of the experiments, when the environment is still novel, autoreceptor KOs of the current study did not display a decrease in locomotor activity. This implies that novelty might play a role in the regulation of activity by *Drd2*. An explanation for the contrast between the effects of D2R autoreceptor deletion on social behavior and activity might be the use of a, long-term, germline intervention, leading to changes in the dopaminergic pathway. Considering the serotonergic heteroreceptor, KO animals showed very subtle decreases in sniffing behavior and approach behavior, and a very subtle increase in locomotor activity, all at specific timepoints. The absence of a significant overall effect on any of the behaviors, suggests only a minor, if any, impact of the deletion of this receptor compared to the autoreceptor (Fig. 4A-C). This coincides with the observed lack of significant changes at specific timepoints regarding sniffing behavior and the relatively small impact on approach behavior and activity. It is unclear why the serotonergic D2R has little to no impact on social behavior or activity. There might, however, be redundancy in this pathway, that compensates for the loss of this heteroreceptor. Dopamine release could be under the control of the D2R autoreceptor, but further transduction of the dopaminergic signal might be mediated postsynaptically by different receptors. Future studies may shed light on the postsynaptic handling of the signal by making use of the genetic tools available in *Drosophila*, as a first step.

The results above show that, by using a continuous long-term assessment of social behaviors in a semi-natural group-housing environment, it is possible to provide a more extensive view on sociability. This approach allows for measuring experimental effects both under (social) novelty and after the animals are familiarized with the environment, and both during their active and their resting phase. It also provides the opportunity to look at the received (i.e. passive) social behavior as the social environment was balanced across treatments. Interestingly, the received social behavior (ie. being sniffed and/or approached) is in line with the active social behavior displayed by both the auto- and heteroreceptor KO animals, although the exact timing of the effect differs from the social behaviors initiated by the focal animal (see figures S17-S20).

However, the used method was subjected to technical constraints, as the software only allowed for the detection of a limited set of social behaviors, from which a selection was made. Aggressive behaviors, for example, could not be measured directly, although aggression has previously been related to D2R (see for example [80]). The measured behaviors, and their automatic registration, were established in Peleh et al. (2020) [65] and consisted of threshold-based algorithms using angle, distance and time as input (see supplementary table S1.).

As both the autoreceptor and heteroreceptor KO mice do not differ from wildtypes regarding social measures on day one, when both the environment and their conspecifics are still unfamiliar, autoreceptor KO mice showed a clear sociability phenotype in the following days, in contrast to the heteroreceptor KO mice. Therefore, the effects seen in this study would, most probably, not have been picked up with a conventional short-term sociability test such as the three-chambered test. Especially if this test had been conducted during the light phase, as the social phenotypes within this study are primarily found in the dark phase, the habitual activity phase of this nocturnal species. Another example of the strength of this studies’ longitudinal approach is the intraindividual comparison between social behavior 24 hours before and after injection of D2R (ant)agonists. Using this method, the acute effects of Sumanirole and L-741,626 were studied in wildtype mice. The D2R agonist Sumanirole decreases sniffing behavior during the first nine hours after injection and decreases the time the treated animals spent on approaching their conspecifics during the first three hours, when compared to vehicle-treated mice (Fig. 5A-B). The decrease in sociability is in line with the results of previous studies using Quinpirole, another D2R agonist (see for example [44]). However, in contrast to those studies, Sumanirole did not affect locomotor activity. Previous research with Sumanirole has shown the agonist to increase activity, although only in models of movements disorders and at higher dosages [81, 82]. As the results of agonistic treatment with Sumanirole, in the current study, are confined to social behavior and deletion of the autoreceptor led to opposite social effects, we can make the caution conclusion that Sumanirole exerts its social effects mainly through the autoreceptor. The absence of an effect on locomotion might be explained by the lack of long-term effects after an acute dosage, or the affinity for either the auto- or heteroreceptor at this dosage. In contrast to the actions of the agonist Sumanirole, antagonism of D2R by L-741,626 does not affect any of the assessed behaviors (Fig. 6A-B). The most simple explanation for this is that the used dosage is insufficient to exert any effects, though behavioral effects were previously found after treatment with a similar dose [60]. Discrepancies from previous data in the results from antagonist treatment could also be explained by the lack of novelty-induced behavior since treated animals are always familiar with the environment.

Although the human genetic target was derived from both males and females [19], this study only assessed male mice. The female estrus cycle adds a layer of complexity [83]. The day of the estrus is indicated to affect behaviors in colonies consisting of both males and females [84], but not much is known about its effect in female-only colonies. As the first study that examines the longitudinal effect of D2R on social functioning, this added complexity might have diluted results and was thus not included. Future studies may include separate observational arenas with only females or create even more complex social dynamics by combining males and females in a single arena (see for example [85]).

The genetic results in mice indicate that not D2 receptors in general, but primarily the autoreceptor is responsible for the modulation of sociability and thereby social withdrawal. These social effects do not overlap with effects on locomotor activity and are, in fact, in the opposite direction. This suggests that the social effects of the D2R autoreceptor are independent of the receptor effects on activity levels. Combining the genetic and pharmacological data proposes a negative correlation between the D2R autoreceptor and sociability, where decreased autoreceptor sensitivity leads to an overly social phenotype and increased autoreceptor sensitivity leads to pathological social withdrawal. This connects the negative symptoms of SZ to the D2R autoreceptor. Furthermore, the positive correlation between autoreceptor sensitivity and locomotor activity might be related to the positive symptoms of SZ, as locomotor activity in rodents is often used as a proxy for these symptoms in rodents [86, 87].

Evidence points towards SZ patients having an increased density of D2R [88]. Further, the most effective treatment against negative symptoms in SZ, the substituted benzamide family of antipsychotics, primarily targets the D2R autoreceptor as an antagonist [52, 53]. This preference might be because of higher affinity for one the isoforms of D2R [52], as Dopamine D2 receptors can be expressed as two different splicing variants, the short isoform (D2RS) and long the isoform (D2RL) [89, 90]. Here, D2RS is suggested to primarily function as the autoreceptor and D2RL as the heteroreceptor [91, 92]. Interestingly, patients suffering from SZ have been shown to display an increase in D2RS relative to D2RL [93], suggesting an overexpression of, primarily, the autoreceptor.

## Conclusion

The results from this study demonstrate that D2R signaling is an important biological substrate for social functioning of distant animal species. This suggests that the genetic molecular building blocks underlying sociability are conserved across evolution. As such, distant species that are highly amenable to genetic manipulations -like *D. melanogaster-* may provide an ideal toolkit for fast genetic modifications and may be used for future screening of human risk genes for neuropsychiatric disorders. When a risk gene shows effects in *D. melanogaster*, this gene can then be selectively manipulated in rodents for a more detailed analysis of its phenotypic effects. This study showed that both the effects of genetic and pharmacological manipulation can be scrutinized in a semi-natural environment employing automated behavioral measurements. Using such a computational ethological approach, this study was able to demonstrate that D2R, and in particular activation of the mammalian autoreceptor, is necessary for sociability, contributing towards a possible intervention target of excessive social withdrawal.

### Supplementary information

Supplementary information

## Data Availability

The datasets generated and/or analyzed during the current study are available from the corresponding author on reasonable request.
