# Supplementary material for: The human neuropsychiatric risk gene Drd2 is necessary for social functioning across evolutionary distant species
Source: Mol Psychiatry. 2023 Dec 19;29(2):518–28. doi: 10.1038/s41380-023-02345-z (PMC11116113; doi:10.1038/s41380-023-02345-z)
Supplement: Supplementary file 1 — Supplementary information [file 41380_2023_2345_MOESM1_ESM.docx]

**Supplementary information**

*Table S1. Definition and key parameters in the algorithm for the automatic analysis of the social behaviors*

|  |  |
| --- | --- |
| ***Sniffing:*** The nose of the mouse is in close proximity to a conspecific | |
| Distance between mice | < 3.5 cm |
| Moving direction (angle) of mouse 1 | < 45° |
| Minimum duration | 0.33 s |
| ***Approach:*** The mouse moves towards a conspecific | |
| Distance between mice | < 100 cm |
| Moving direction (angle) of mouse 1 | < 45° |
| Distance to be traveled by mouse 1 towards mouse 2 | > 7 cm |
| Velocity of mouse 1 | > 4 cm/s |
|  |  |
|  |  |
|  |  |


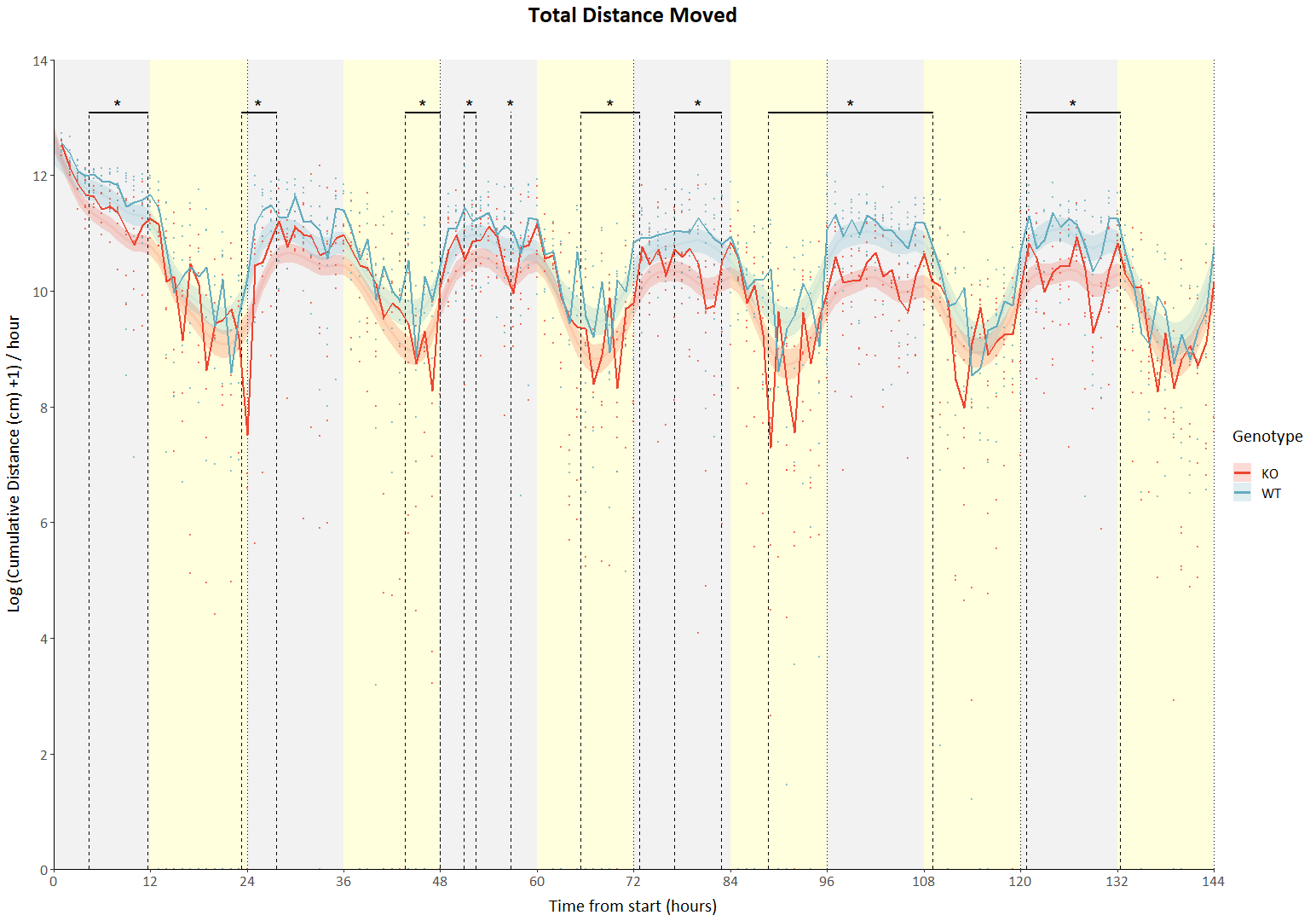


*Figure S1. Drd2 autoreceptor KO mice: predicted locomotor activity based on modeled data, and mean of observed values.*

*Data is presented as the logarithm of cumulative total distance moved in centimeters on the Y-axis (mean±SEM) based on 1-hour bins, with time from start of the experiment on the X-axis in hours. Autoreceptor knockout animals (n=7) are shown as the red lines and dots, wildtype animals (n=7) are shown as blue lines and dots. Yellow shading behind the graph indicates the light phase, where gray shading indicates the dark phase. n=7; * p<0,05 based on difference plots after modeling the data with a GAM.*


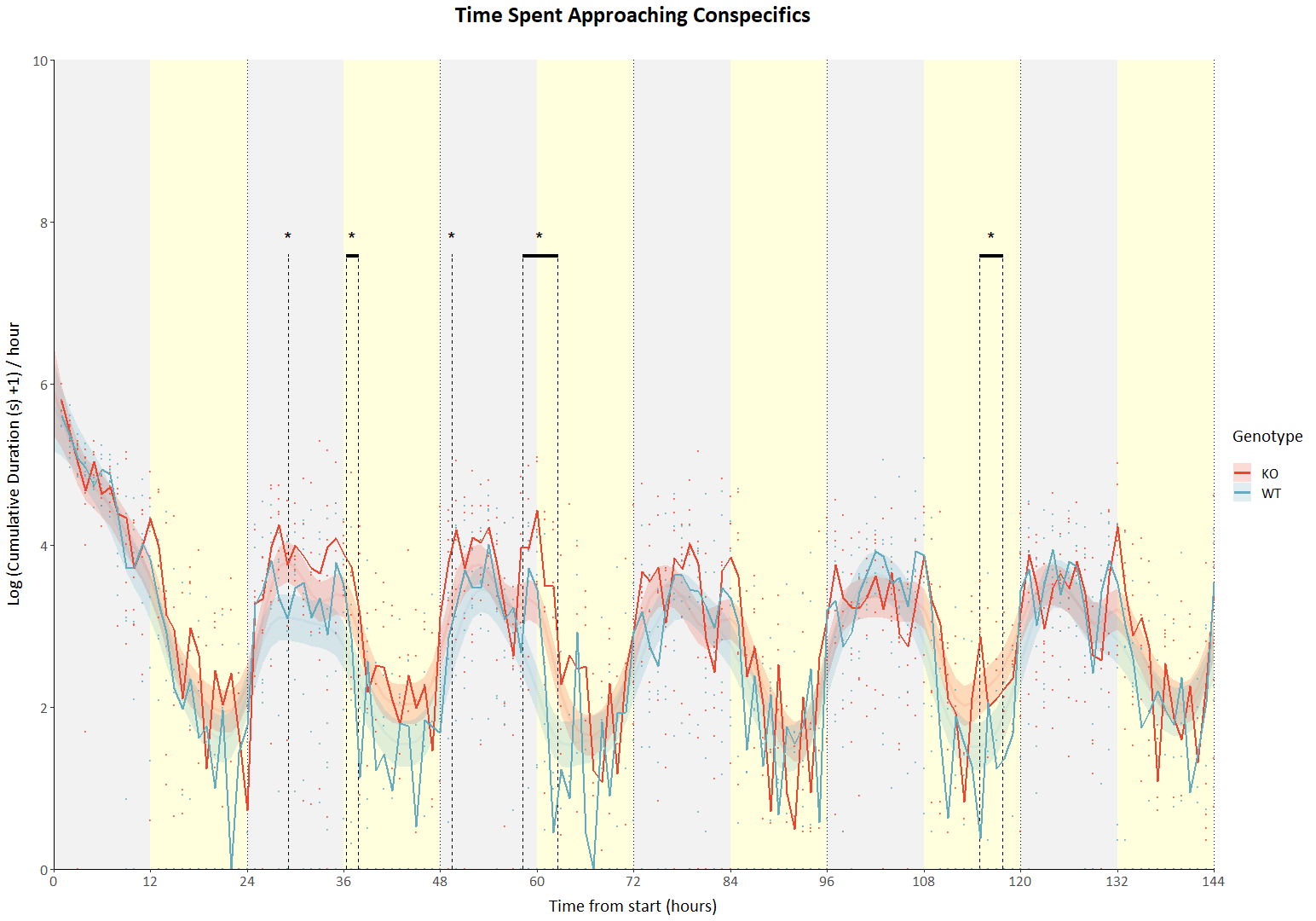


*Figure S2. Drd2 autoreceptor KO mice: predicted approach behavior based on modeled data, and mean of observed values.*

*Data is presented as the logarithm of cumulative time spent approaching conspecifics in seconds on the Y-axis (mean±SEM) based on 1-hour bins, with time from start of the experiment on the X-axis in hours. Autoreceptor knockout animals (n=7) are shown as the red lines and dots, wildtype animals (n=7) are shown as blue lines and dots. Yellow shading behind the graph indicates the light phase, where gray shading indicates the dark phase. n=7; * p<0,05 based on difference plots after modeling the data with a GAM.*


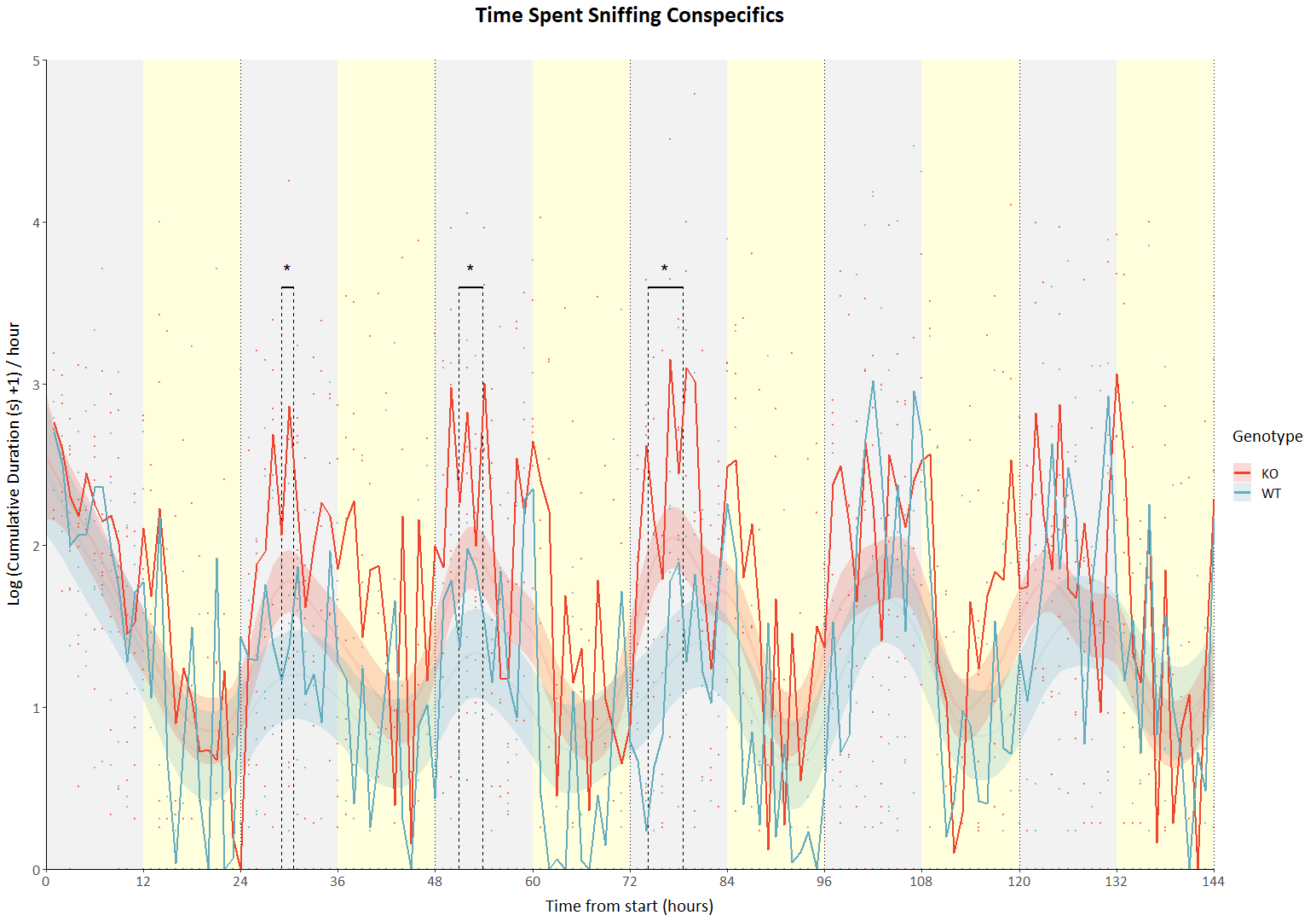


*Figure S3. Drd2 autoreceptor KO mice: predicted sniffing behavior based on modeled data, and mean of observed values.*

*Data is presented as the logarithm of cumulative time spent sniffing conspecifics in seconds on the Y-axis (mean±SEM) based on 1-hour bins, with time from start of the experiment on the X-axis in hours. Autoreceptor knockout animals (n=7) are shown as the red lines and dots, wildtype animals (n=7) are shown as blue lines and dots. Yellow shading behind the graph indicates the light phase, where gray shading indicates the dark phase. n=7; * p<0,05 based on difference plots after modeling the data with a GAM.*


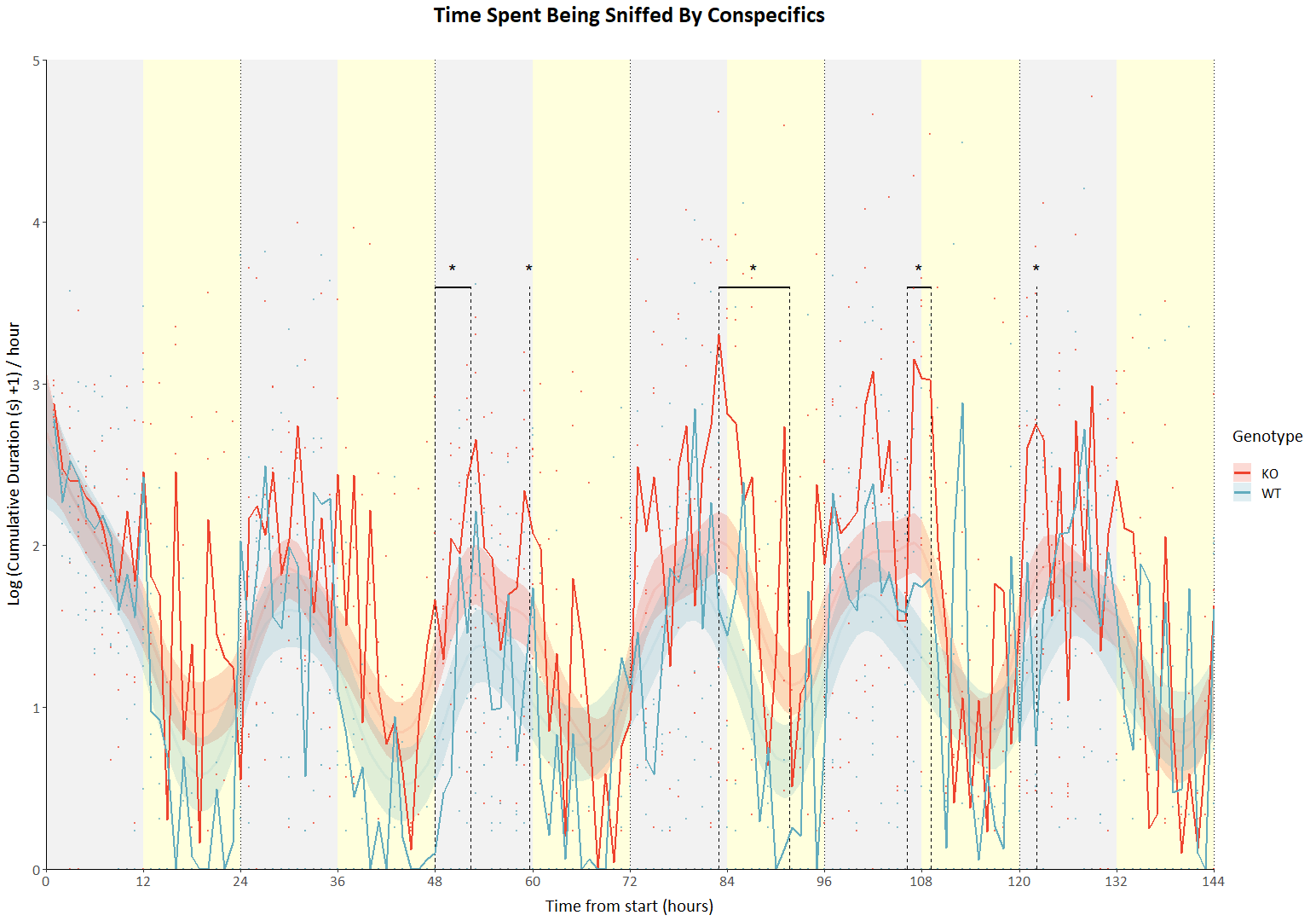


*Figure S4. Drd2 autoreceptor KO mice: predicted passive sniffing behavior based on modeled data, and mean of observed values.*

*Data is presented as the logarithm of cumulative time spent being sniffed by conspecifics in seconds on the Y-axis (mean±SEM) based on 1-hour bins, with time from start of the experiment on the X-axis in hours. Autoreceptor knockout animals (n=7) are shown as the red lines and dots, wildtype animals (n=7) are shown as blue lines and dots. Yellow shading behind the graph indicates the light phase, where gray shading indicates the dark phase. n=7; * p<0,05 based on difference plots after modeling the data with a GAM.*


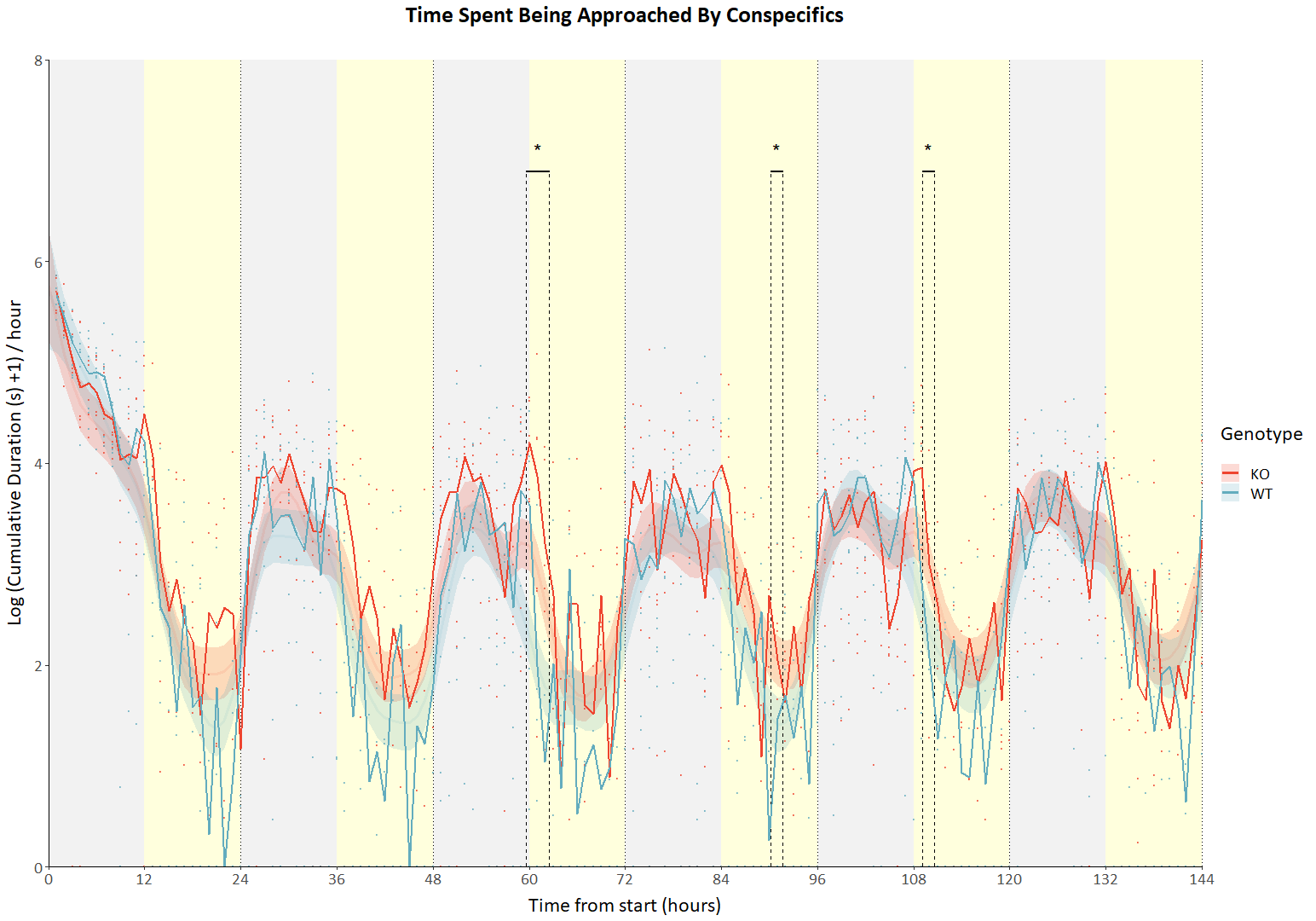


*Figure S5. Drd2 autoreceptor KO mice: predicted passive approach behavior based on modeled data, and mean of observed values.*

*Data is presented as the logarithm of cumulative time spent being approached by conspecifics in seconds on the Y-axis (mean±SEM) based on 1-hour bins, with time from start of the experiment on the X-axis in hours. Autoreceptor knockout animals (n=7) are shown as the red lines and dots, wildtype animals (n=7) are shown as blue lines and dots. Yellow shading behind the graph indicates the light phase, where gray shading indicates the dark phase. n=7; * p<0,05 based on difference plots after modeling the data with a GAM.*


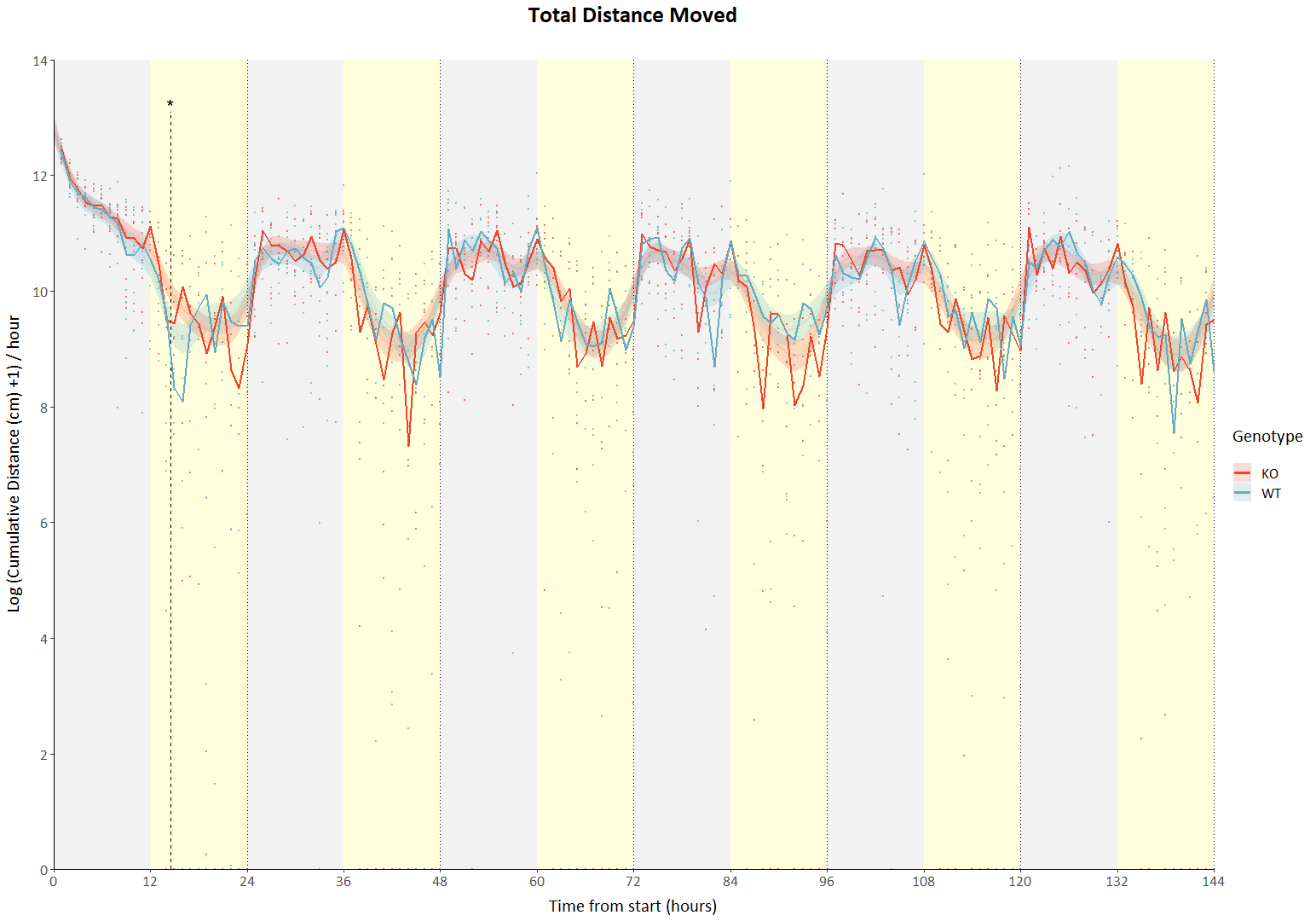


*Figure S6. Serotonergic Drd2 heteroreceptor KO mice: predicted locomotor activity based on modeled data, and mean of observed values.*

*Data is presented as the logarithm of cumulative total distance moved in centimeters on the Y-axis (mean±SEM) based on 1-hour bins, with time from start of the experiment on the X-axis in hours. Autoreceptor knockout animals (n=7) are shown as the red lines and dots, wildtype animals (n=7) are shown as blue lines and dots. Yellow shading behind the graph indicates the light phase, where gray shading indicates the dark phase. n=7; * p<0,05 based on difference plots after modeling the data with a GAM.*


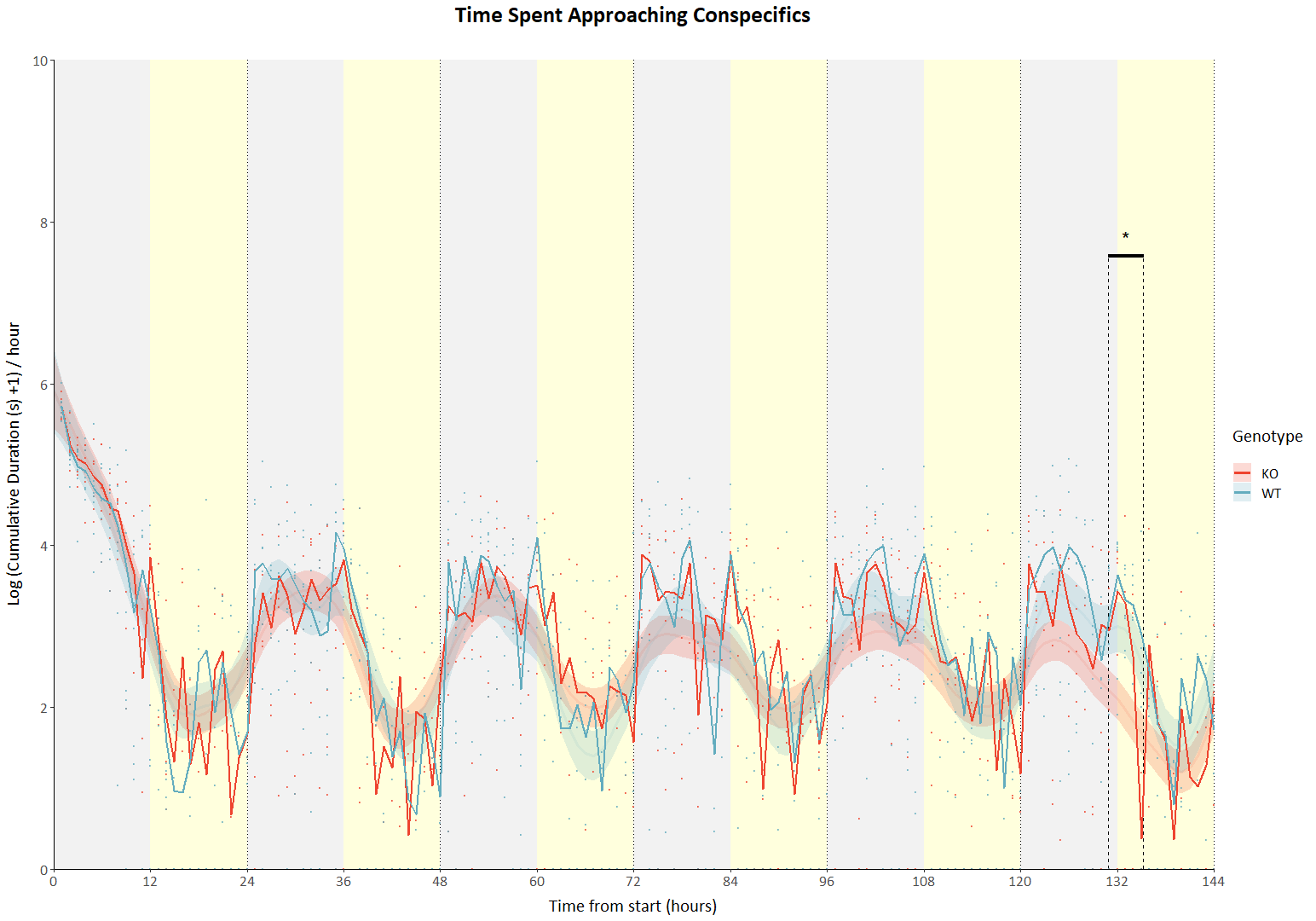


*Figure S7. Serotonergic Drd2 heteroreceptor KO mice: predicted approach behavior based on modeled data, and mean of observed values.*

*Data is presented as the logarithm of cumulative time spent approaching conspecifics in seconds on the Y-axis (mean±SEM) based on 1-hour bins, with time from start of the experiment on the X-axis in hours. Autoreceptor knockout animals (n=7) are shown as the red lines and dots, wildtype animals (n=7) are shown as blue lines and dots. Yellow shading behind the graph indicates the light phase, where gray shading indicates the dark phase. n=7; * p<0,05 based on difference plots after modeling the data with a GAM.*


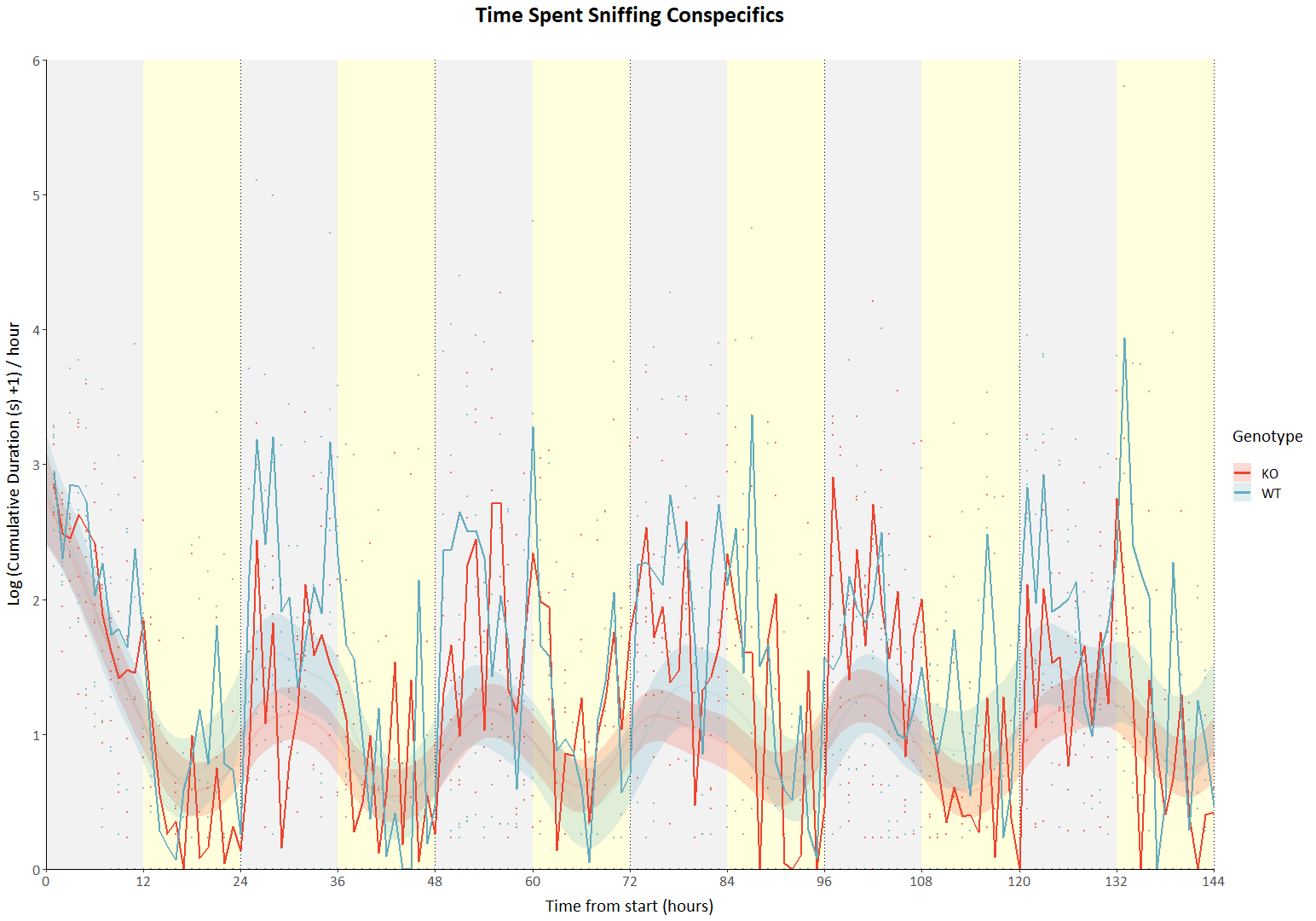


*Figure S8. Serotonergic Drd2 heteroreceptor KO mice: predicted sniffing behavior based on modeled data, and mean of observed values.*

*Data is presented as the logarithm of cumulative time spent sniffing conspecifics in seconds on the Y-axis (mean±SEM) based on 1-hour bins, with time from start of the experiment on the X-axis in hours. Autoreceptor knockout animals (n=7) are shown as the red lines and dots, wildtype animals (n=7) are shown as blue lines and dots. Yellow shading behind the graph indicates the light phase, where gray shading indicates the dark phase. n=7; * p<0,05 based on difference plots after modeling the data with a GAM.*


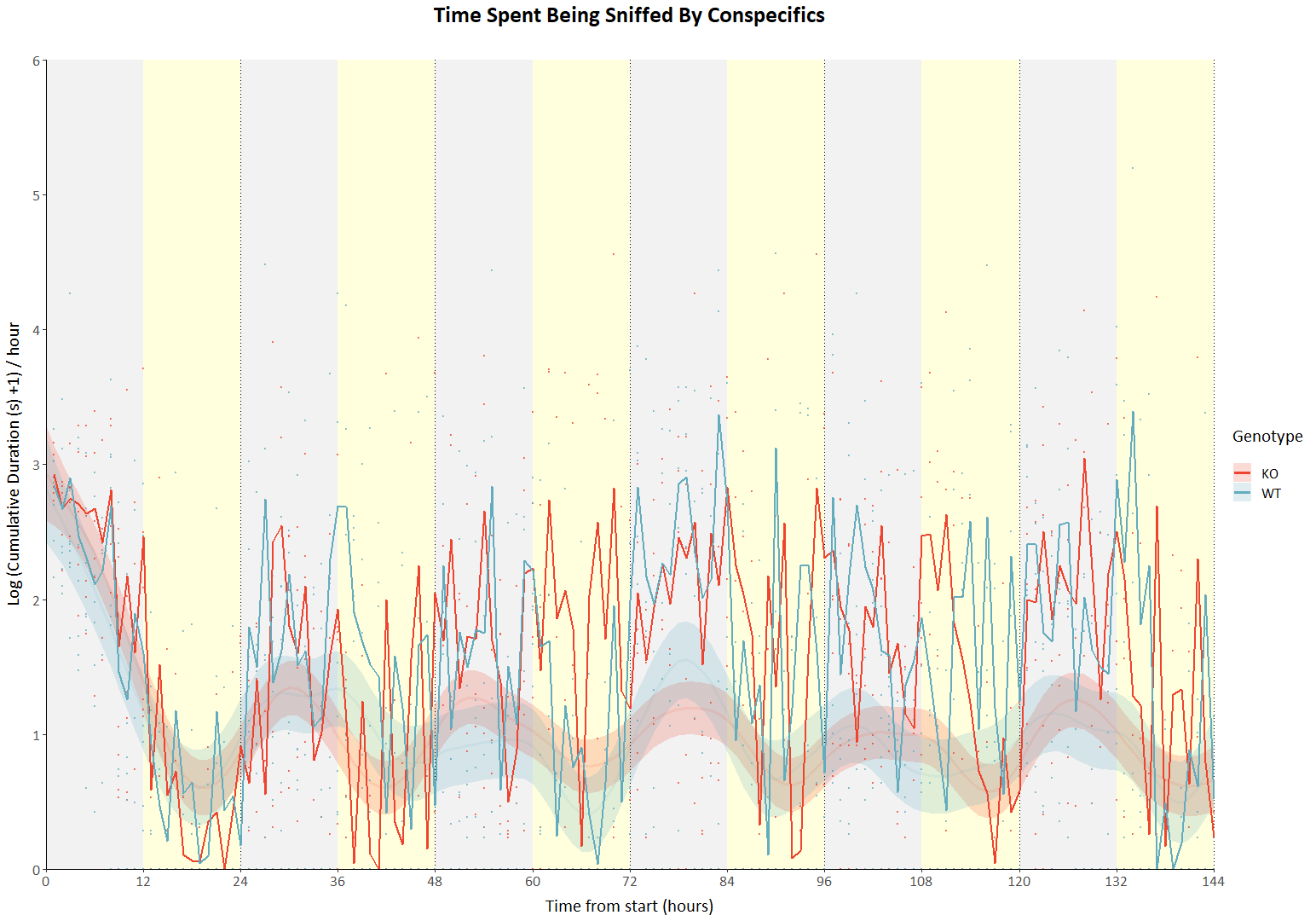


*Figure S9. Serotonergic Drd2 heteroreceptor KO mice: predicted passive sniffing behavior based on modeled data, and mean of observed values.*

*Data is presented as the logarithm of cumulative time spent being sniffed by conspecifics in seconds on the Y-axis (mean±SEM) based on 1-hour bins, with time from start of the experiment on the X-axis in hours. Autoreceptor knockout animals (n=7) are shown as the red lines and dots, wildtype animals (n=7) are shown as blue lines and dots. Yellow shading behind the graph indicates the light phase, where gray shading indicates the dark phase. n=7; * p<0,05 based on difference plots after modeling the data with a GAM.*


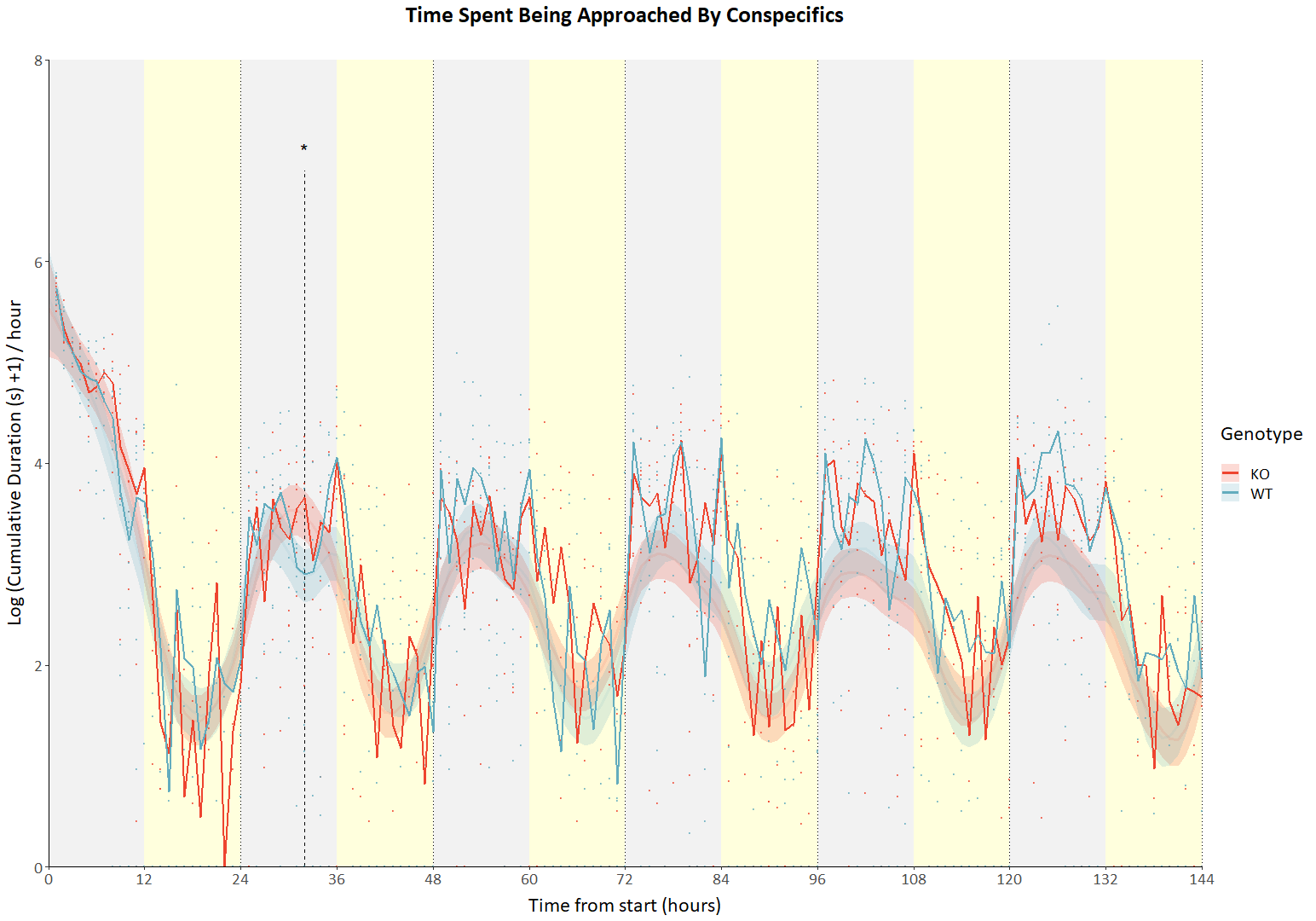


*Figure S10. Serotonergic Drd2 heteroreceptor KO mice: predicted passive approach behavior based on modeled data, and mean of observed values.*

*Data is presented as the logarithm of cumulative time spent being approached by conspecifics in seconds on the Y-axis (mean±SEM) based on 1-hour bins, with time from start of the experiment on the X-axis in hours. Autoreceptor knockout animals (n=7) are shown as the red lines and dots, wildtype animals (n=7) are shown as blue lines and dots. Yellow shading behind the graph indicates the light phase, where gray shading indicates the dark phase. n=7; * p<0,05 based on difference plots after modeling the data with a GAM.*


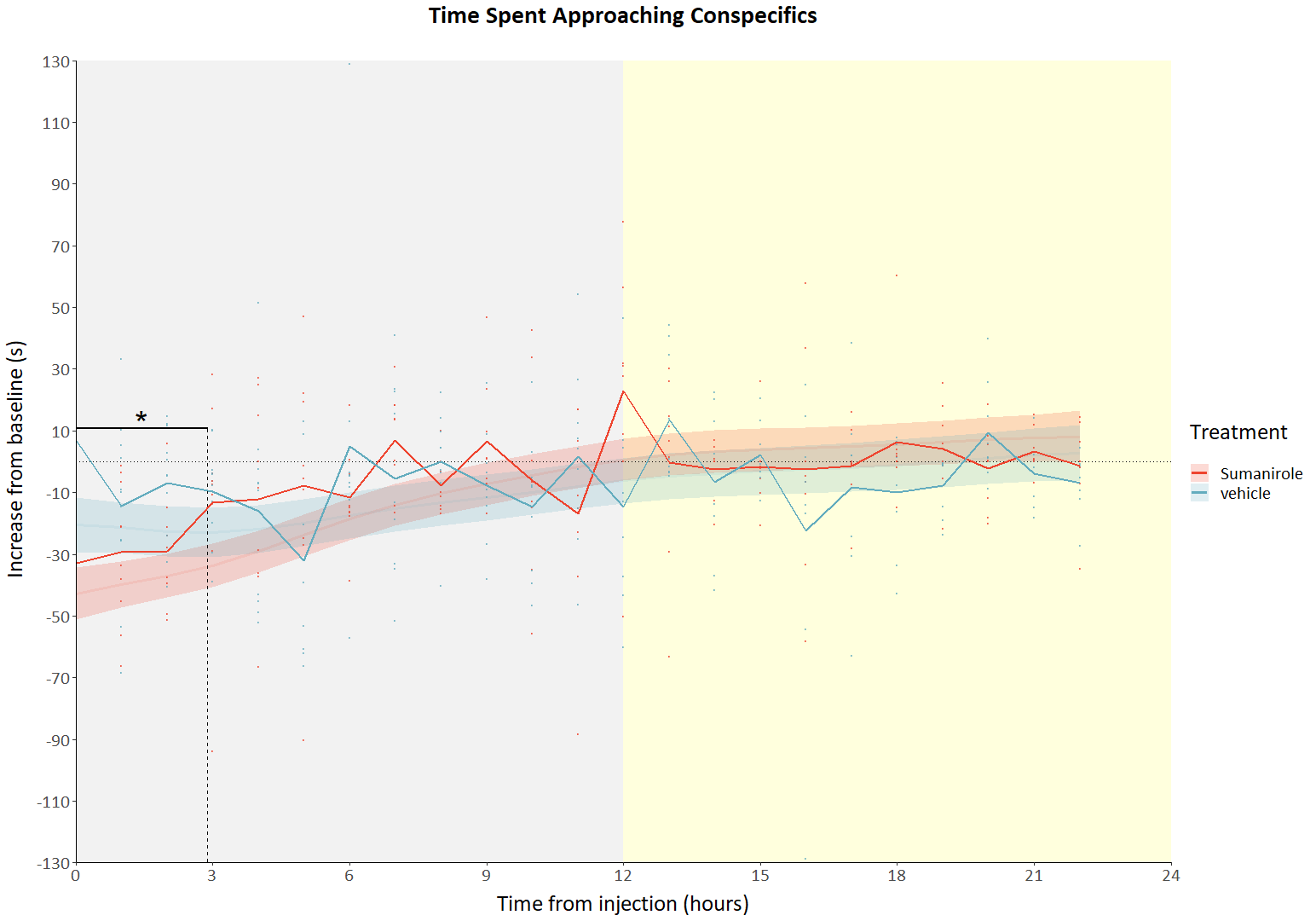


*Figure S11. Mice injected with the D2R agonist Sumanirole: predicted approach behavior based on modeled data, and mean of observed values.*

*Data is presented as the increase in time spent approaching conspecifics compared to baseline on the Y-axis (mean±SEM) based on 1-hour bins, with time from injection on the X-axis in hours. Sumanirole-treated animals (n=9) are shown as the red lines and dots, vehicle-treated animals (n=9) are shown as blue lines and dots. Yellow shading behind the graph indicates the light phase, where gray shading indicates the dark phase. * p<0,05 based on difference plots after modeling the data with a GAM.*


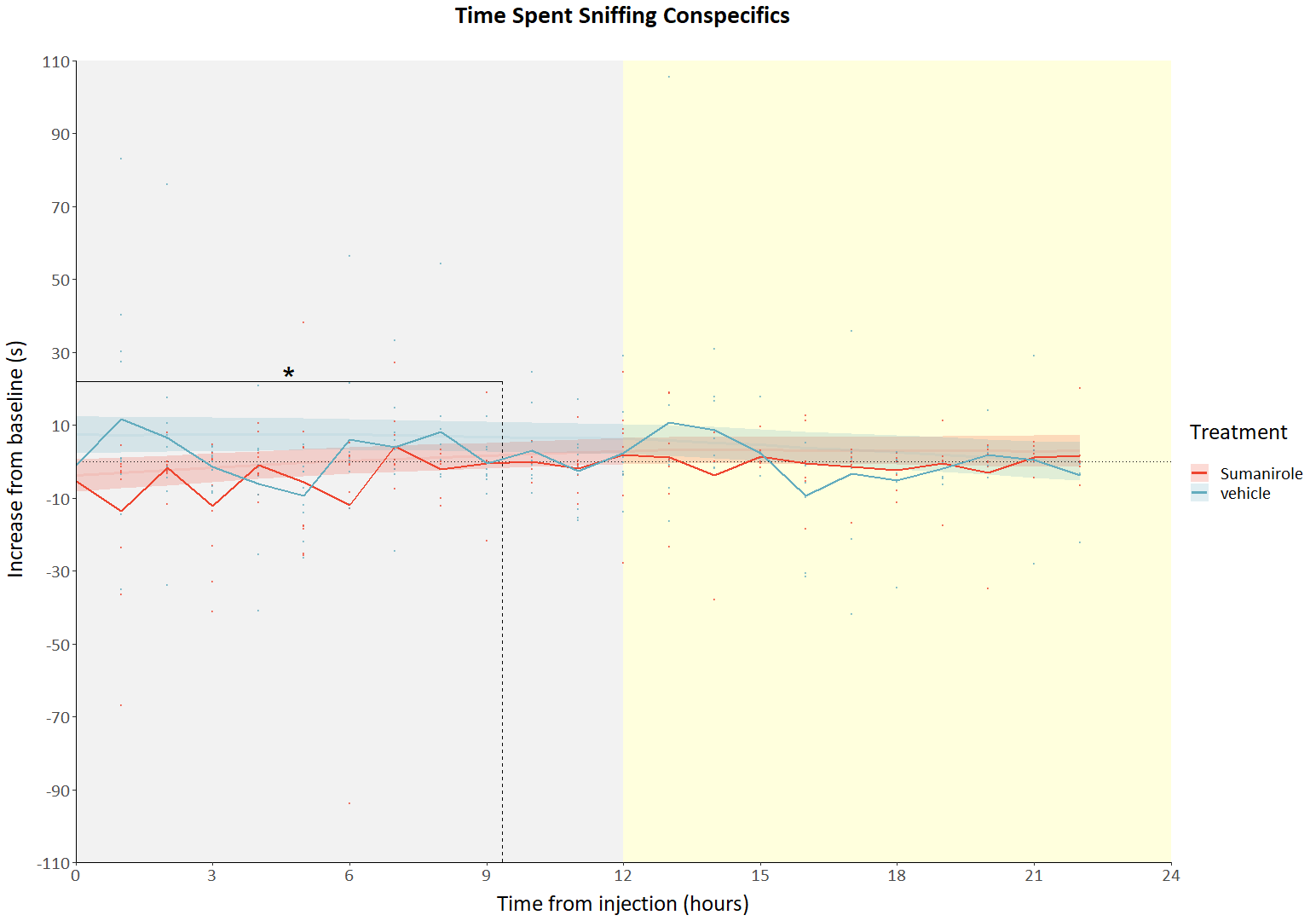


*Figure S12. Mice injected with the D2R agonist Sumanirole: predicted sniffing behavior based on modeled data, and mean of observed values.*

*Data is presented as the increase in time spent sniffing conspecifics compared to baseline on the Y-axis (mean±SEM) based on 1-hour bins, with time from injection on the X-axis in hours. Sumanirole-treated animals (n=9) are shown as the red lines and dots, vehicle-treated animals (n=9) are shown as blue lines and dots. Yellow shading behind the graph indicates the light phase, where gray shading indicates the dark phase. * p<0,05 based on difference plots after modeling the data with a GAM.*


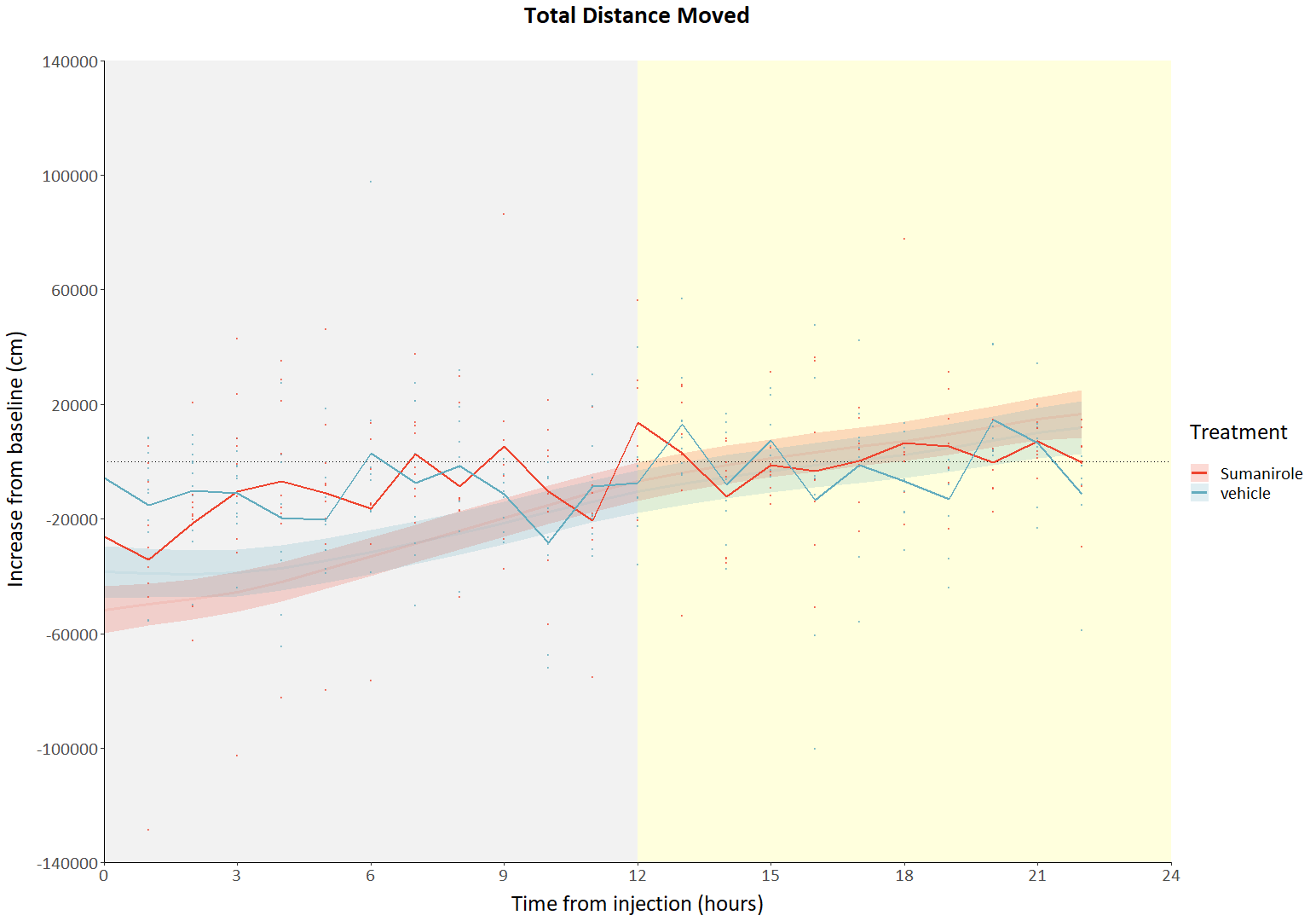


*Figure S13. Mice injected with the D2R agonist Sumanirole: predicted locomotor activity based on modeled data, and mean of observed values.*

*Data is presented as the increase in total distance moved compared to baseline on the Y-axis (mean±SEM) based on 1-hour bins, with time from injection on the X-axis in hours. Sumanirole treated animals (n=9) are shown as the red lines and dots, vehicle-treated animals (n=9) are shown as blue lines and dots. Yellow shading behind the graph indicates the light phase, where gray shading indicates the dark phase.*


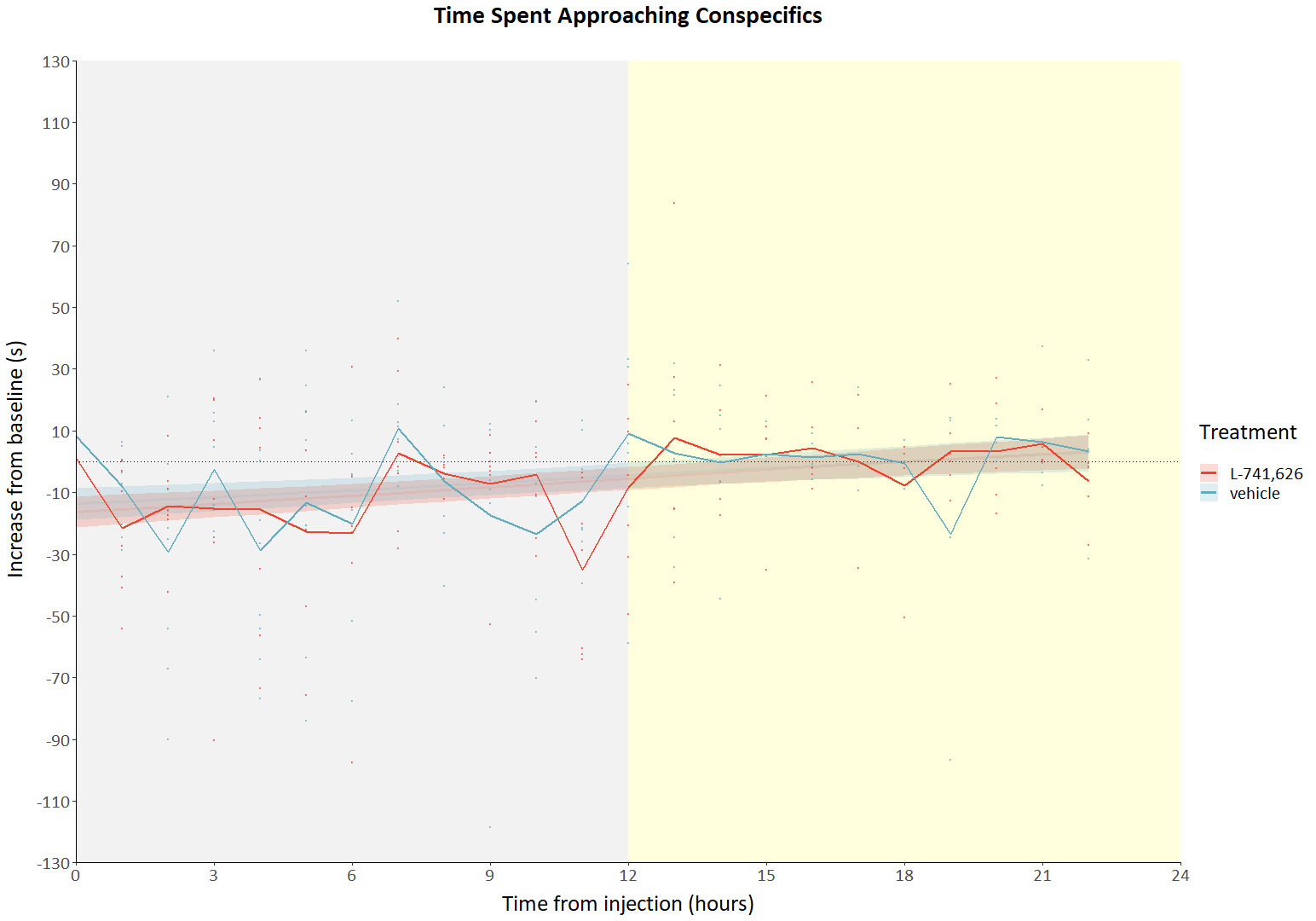


*Figure S14. Mice injected with the D2R antagonist L-741,626: predicted approach behavior based on modeled data, and mean of observed values.*

*Data is presented as the increase in time spent approaching conspecifics compared to baseline on the Y-axis (mean±SEM) based on 1-hour bins, with time from injection on the X-axis in hours. L-741,626 treated animals (n=8) are shown as the red lines and dots, vehicle-treated animals (n=9) are shown as blue lines and dots. Yellow shading behind the graph indicates the light phase, where gray shading indicates the dark phase.*


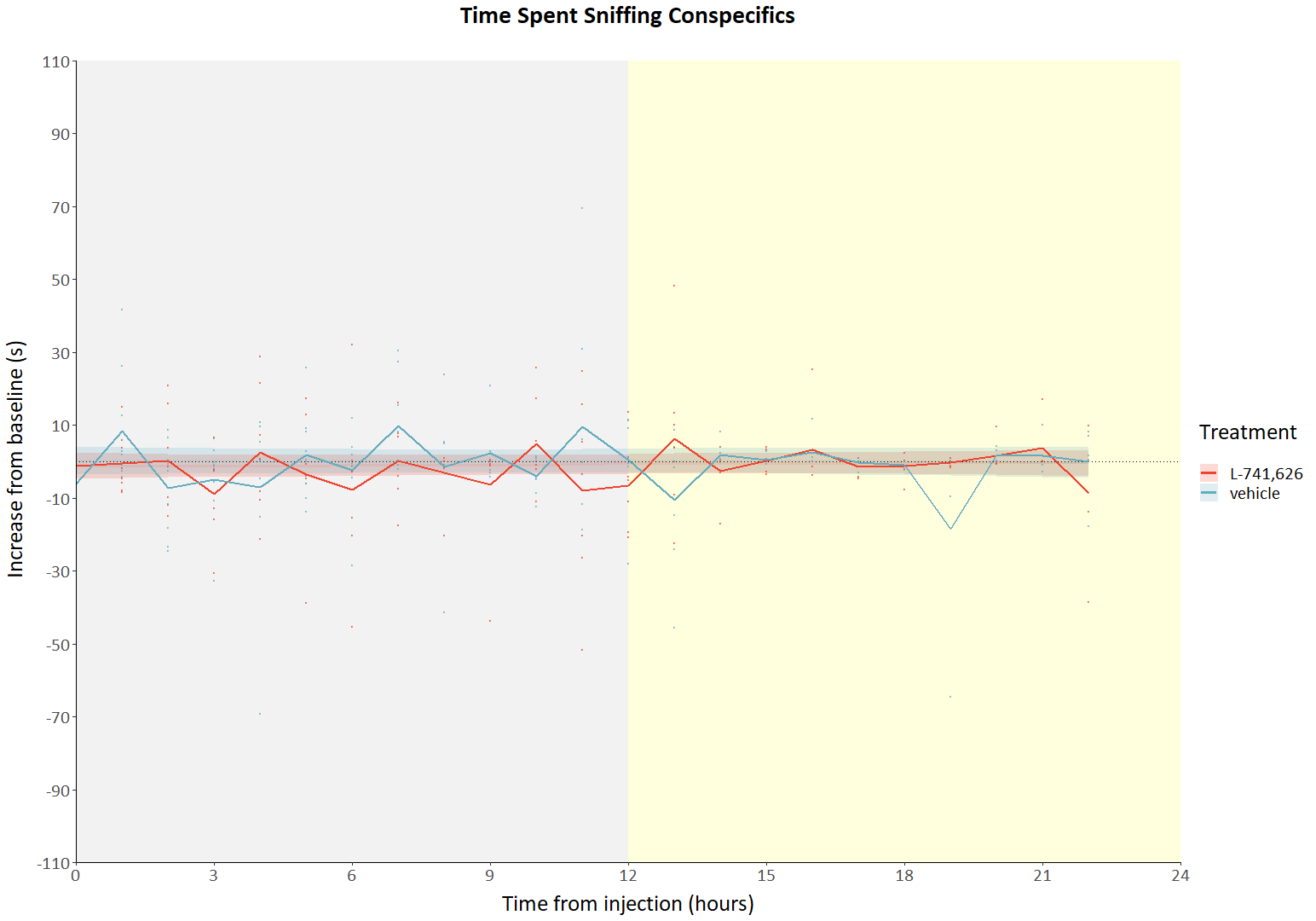


*Figure S15. Mice injected with the D2R antagonist L-741,626: predicted sniffing behavior based on modeled data, and mean of observed values.*

*Data is presented as the increase in time spent sniffing conspecifics compared to baseline on the Y-axis (mean±SEM) based on 1-hour bins, with time from injection on the X-axis in hours. L-741,626 treated animals (n=8) are shown as the red lines and dots, vehicle-treated animals (n=9) are shown as blue lines and dots. Yellow shading behind the graph indicates the light phase, where gray shading indicates the dark phase.*


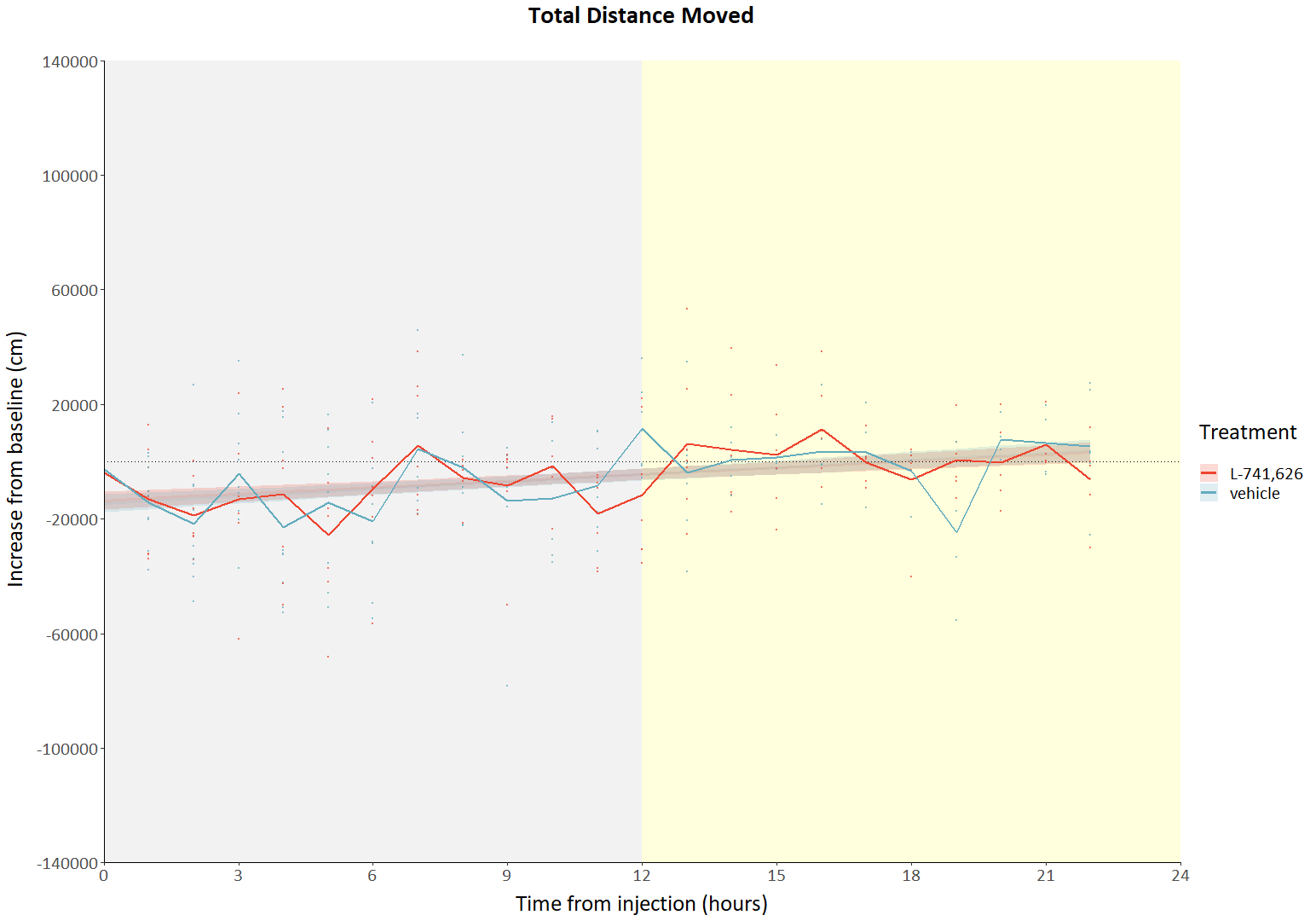


*Figure S16. Mice injected with the D2R antagonist L-741,626: predicted locomotor activity based on modeled data, and mean of observed values.*

*Data is presented as the increase in total distance moved compared to baseline on the Y-axis (mean±SEM) based on 1-hour bins, with time from injection on the X-axis in hours. L-741,626 treated animals (n=8) are shown as the red lines and dots, vehicle-treated animals (n=9) are shown as blue lines and dots. Yellow shading behind the graph indicates the light phase, where gray shading indicates the dark phase.*


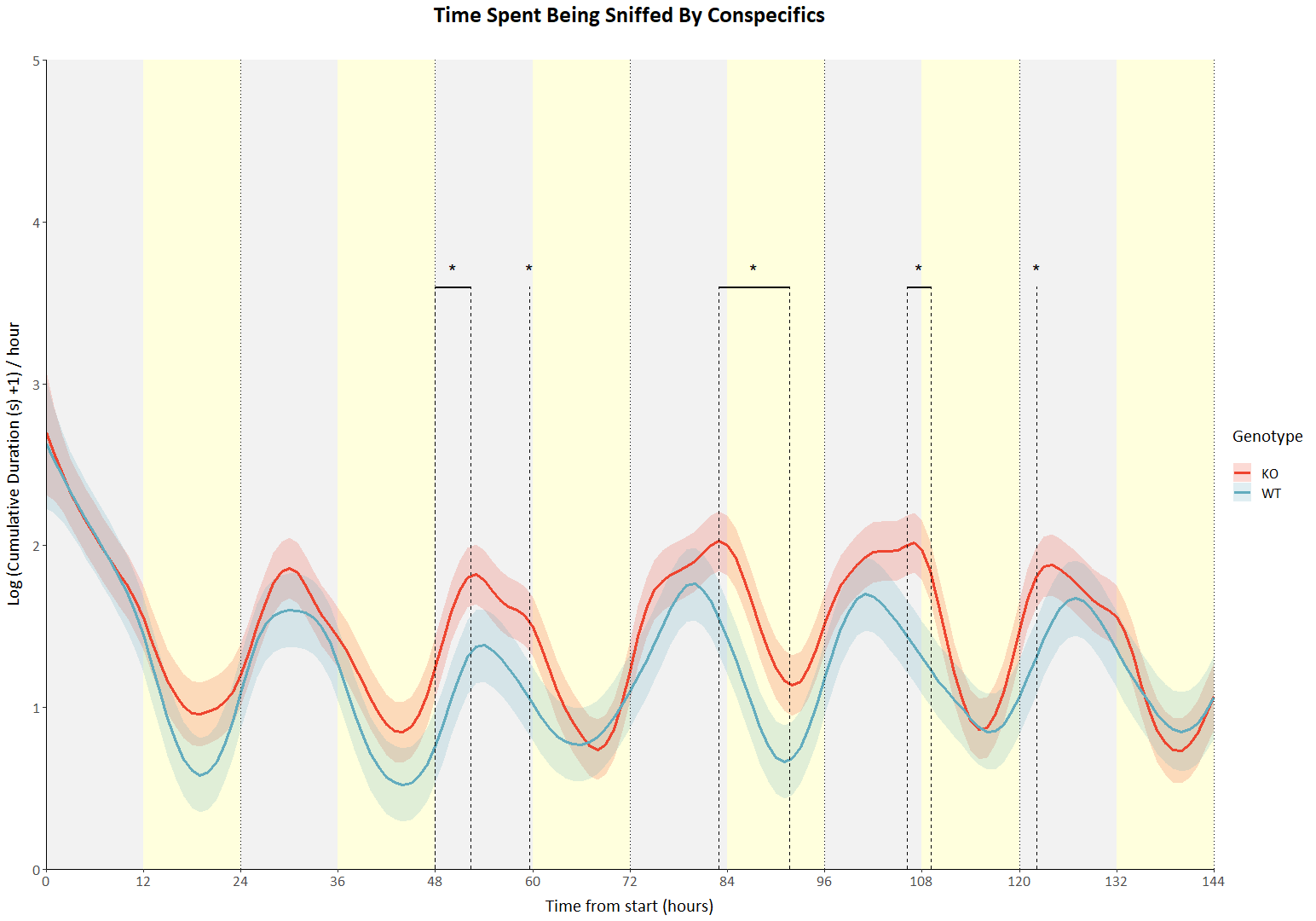


*Figure S17. Drd2 autoreceptor KO mice: predicted passive sniffing behavior based on modeled data.*

*Data is presented as the logarithm of cumulative time spent being sniffed by conspecifics in seconds on the Y-axis (mean±SEM) based on 1-hour bins, with time from start of the experiment on the X-axis in hours. Autoreceptor knockout animals (n=7) are shown as the red lines, wildtype animals (n=7) are shown as blue lines. Yellow shading behind the graph indicates the light phase, where gray shading indicates the dark phase. n=7; * p<0,05 based on difference plots after modeling the data with a GAM. Main effect of genotype P=* *0.046; Genotype*time interaction: P<0.001, F_(24.036, 1907.727)_=* *4.71.*

*Figure S18. Drd2 autoreceptor KO mice: predicted passive sniffing behavior based on modeled data.*
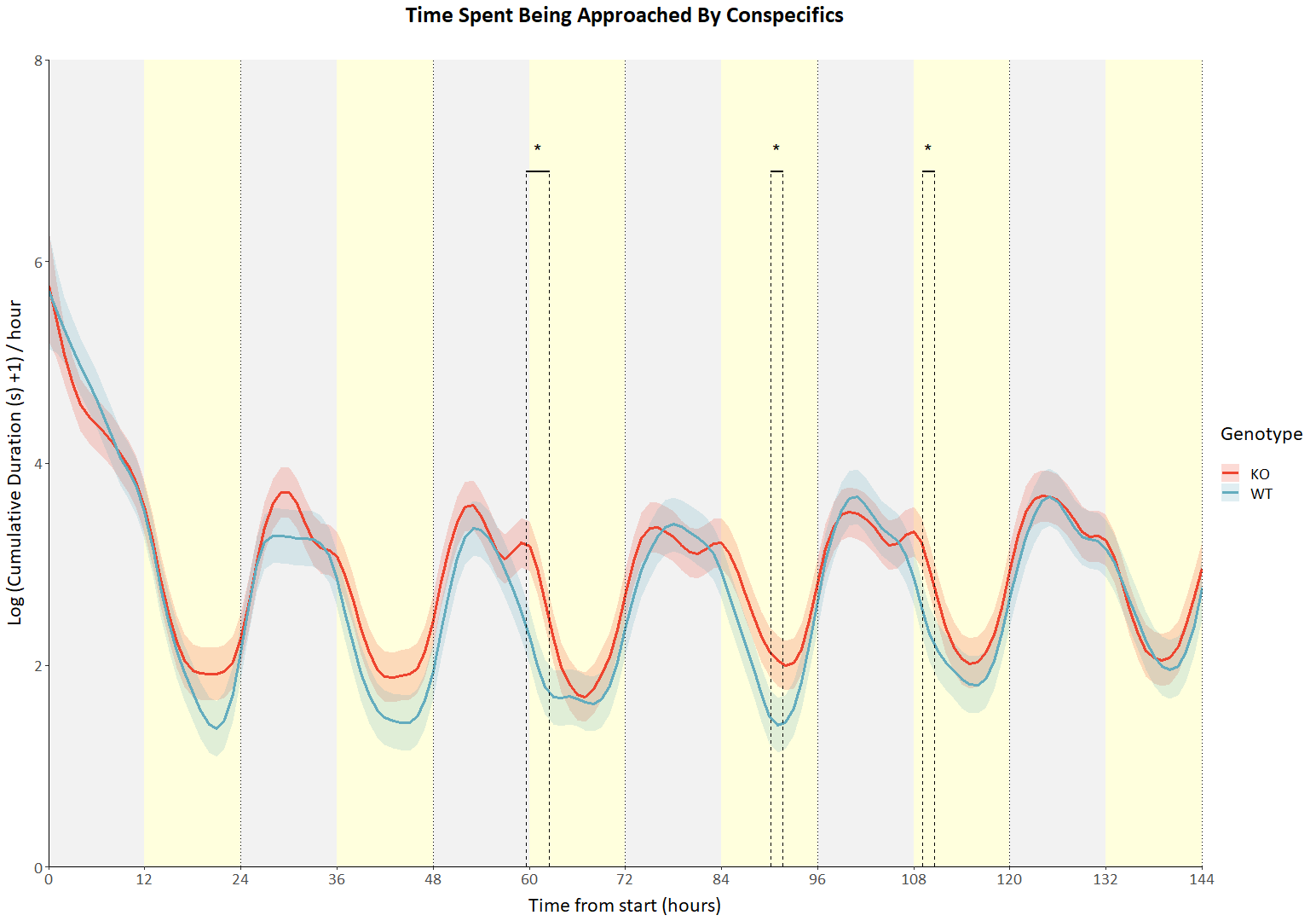


*Data is presented as the logarithm of cumulative time spent being approached by conspecifics in seconds on the Y-axis (mean±SEM) based on 1-hour bins, with time from start of the experiment on the X-axis in hours. Autoreceptor knockout animals (n=6) are shown as the red lines, wildtype animals (n=8) are shown as blue lines. Yellow shading behind the graph indicates the light phase, where gray shading indicates the dark phase. n=7; * p<0,05 based on difference plots after modeling the data with a GAM. Main effect of genotype P=* *0.056; Genotype*time interaction: P<0.001, F_(27.836, 1904.554)_=* *5.77.*


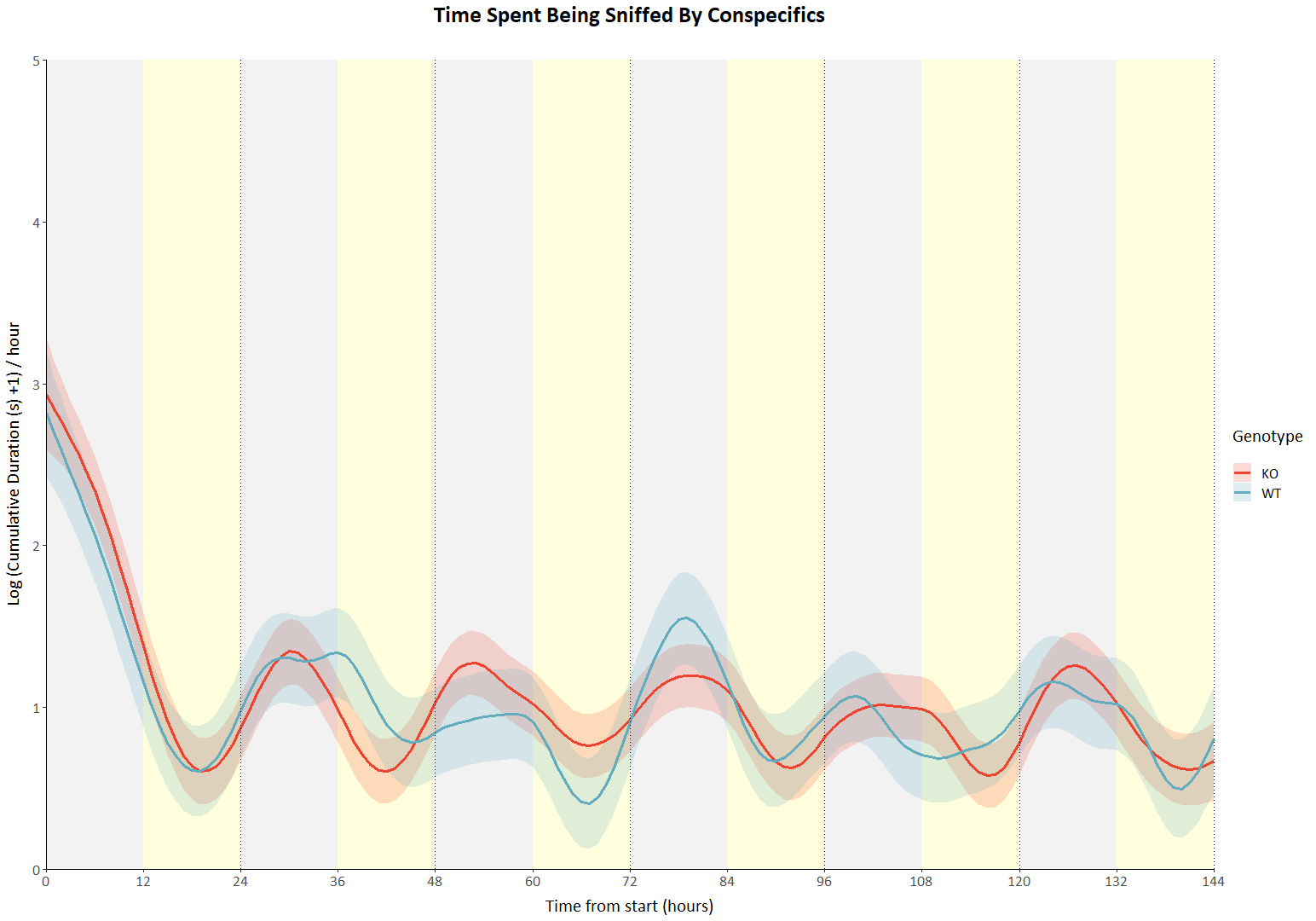


*Figure S19. Serotonergic Drd2 heteroreceptor KO mice: predicted passive sniffing behavior based on modeled data.*

*Data is presented as the logarithm of cumulative time spent being sniffed by conspecifics in seconds on the Y-axis (mean±SEM) based on 1-hour bins, with time from start of the experiment on the X-axis in hours. Autoreceptor knockout animals (n=6) are shown as the red lines, wildtype animals (n=8) are shown as blue lines. Yellow shading behind the graph indicates the light phase, where gray shading indicates the dark phase. n=7; * p<0,05 based on difference plots after modeling the data with a GAM. Main effect of genotype P=* *0.894; Genotype*time interaction: P<0.001, F_(16.608, 1825.156)_=* *2.34.*

*Figure S20. Serotonergic Drd2 heteroreceptor KO mice: predicted passive sniffing behavior based on modeled data.*
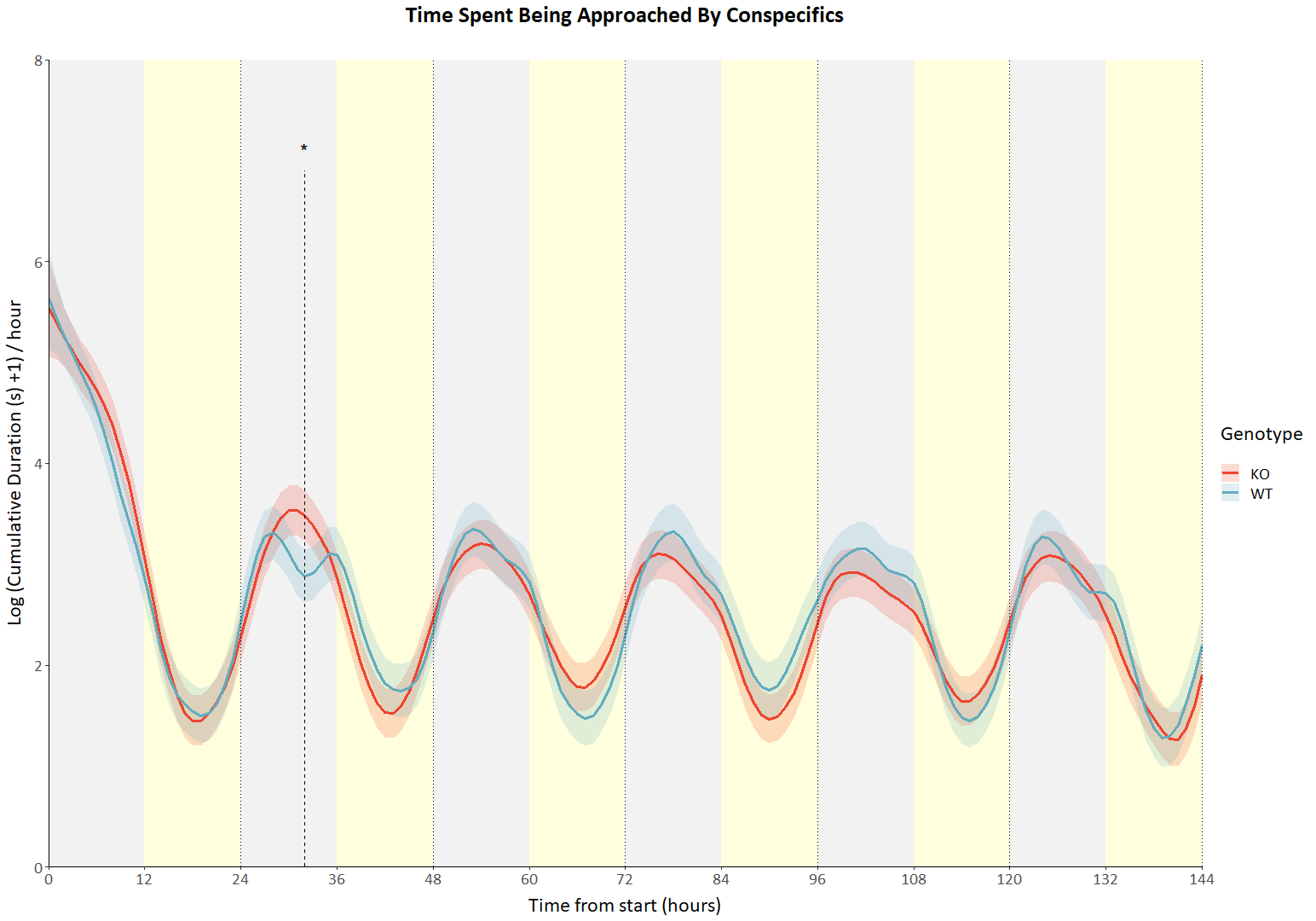


*Data is presented as the logarithm of cumulative time spent being approached by conspecifics in seconds on the Y-axis (mean±SEM) based on 1-hour bins, with time from start of the experiment on the X-axis in hours. Autoreceptor knockout animals (n=7) are shown as the red lines, wildtype animals (n=7) are shown as blue lines. Yellow shading behind the graph indicates the light phase, where gray shading indicates the dark phase. n=7; * p<0,05 based on difference plots after modeling the data with a GAM. Main effect of genotype P=* *0.747; Genotype*time interaction: P<0.001, F_(27.092, 1836.989)_=8.28.*
